# BMAL1 regulates tubular epithelial-derived exosomal miR-27a-3p to inhibit macrophage–myofibroblast transition and alleviate ischemia/reperfusion-induced renal fibrosis

**DOI:** 10.7150/thno.127538

**Published:** 2026-05-18

**Authors:** Wu Chen, Sheng Zhao, Songyuan Yang, Weimin Yu, Ting Rao, Xiangjun Zhou, Yuan Ruan, Fan Zou, Fan Cheng, Wei Li

**Affiliations:** 1 Department of Urology, Renmin Hospital of Wuhan University, Wuhan, 430060, China.; 2 Department of Anesthesiology, Renmin Hospital of Wuhan University, Wuhan, 430060, China.

**Keywords:** renal ischemia‒reperfusion injury, renal fibrosis, brain and muscle ARNT-like 1, exosomes, macrophage-to-myofibroblast transition

## Abstract

**Rationale:**

During ischemia‒reperfusion injury (IRI), *BMAL1* has been shown to alleviate inflammation and kidney damage. However, the function of the tubular epithelium–macrophage interaction mediated by *BMAL1* in IRI-induced renal fibrosis is still unclear.

**Methods:**

A mouse model of kidney-specific *BMAL1* overexpression was developed to study how *BMAL1* affects renal fibrosis, exosome production, and the macrophage-to-myofibroblast transition (MMT). The role of exosomes in the MMT and renal fibrosis was examined in both *in vitro* and *in vivo* studies using exosomes extracted from TCMK-1 cells. Exosomes from *BMAL1*-overexpressing TCMK-1 cells subjected to hypoxia–reoxygenation (H/R) were isolated and subjected to miRNA sequencing to identify key exosomal components. Exosomal *miR-27a-3p* regulation by *BMAL1* and its downstream effects on *TGFBR1/smad3* in macrophages were investigated using a variety of experimental methods. To assess the effect of exosomal *miR-27a-3p* on MMT and renal fibrosis, additional *in vitro* and *in vivo* investigations were conducted.

**Results:**

Renal IRI increased exosome secretion, promoted MMT, and exacerbated renal fibrosis, whereas *BMAL1* overexpression or *Rab27a* knockout significantly attenuated IRI-induced MMT and fibrotic progression. Exosomes derived from H/R-treated tubular epithelial cells further exacerbated MMT and renal fibrosis in an IRI model. Notably, tubular-specific overexpression of *BMAL1*, elevation of exosomal *miR-27a-3p* levels, or inhibition of exosome secretion significantly attenuated the progression of both MMT and fibrosis. Mechanistic studies demonstrated that *BMAL1* binds directly to the *miR-27a-3p* promoter region, enhancing transcription. Exosomal *miR-27a-3p* subsequently targets *TGFBR1* mRNA in macrophages, thereby suppressing the *TGFBR1/smad3* signaling pathway and ultimately attenuating MMT and renal fibrosis.

**Conclusions:**

*BMAL1* expression was suppressed in IRI, which promoted MMT and renal fibrosis via the exosomal* miR-27a-3p*–*TGFBR1/smad3* pathway. Targeting this signaling pathway may offer a potential therapeutic strategy for alleviating IRI-induced renal fibrosis.

## Introduction

One of the primary causes of acute kidney injury (AKI) is renal ischemia‒reperfusion injury (IRI), which frequently occurs in patients with shock, sepsis, kidney transplantation, or postoperative procedures. AKI is a significant risk factor for chronic kidney disease (CKD), which is associated with high morbidity and mortality and mostly presents as a significant decline in kidney function 1-3. One common pathological alteration that occurs as AKI progresses to CKD is renal fibrosis. Accordingly, how to delay and alleviate fibrosis has been the focus of CKD-related research.

Renal IRI involves numerous cellular and molecular changes. After kidney damage occurs, a range of bioactive substances released by renal tubular epithelial cells may promote interstitial fibrosis and inflammatory cell infiltration, which are thought to be essential for the development of kidney disease 4-6. Extracellular vesicles, such as exosomes, have been demonstrated in recent research to be essential bridges of intercellular communication. Exosomes are bilayer vesicles that range in diameter from 30 to 150 nm. They are abundant in proteins and nucleic acids, including circular RNAs, long noncoding RNAs (lncRNAs), and microRNAs (miRNAs) 7-10. According to recent research, exosomes can be secreted by nearly all cells under both physiological and pathological conditions. By delivering bioactive substances such as proteins and nucleic acids to target cells, exosomes can alter the genetic information and biological functions of the recipient cells 11, 12. Polyvesicular transport is involved in exosome secretion, and Rab guanosine triphosphatases, such as *Rab27a* and *Rab27b*, have been demonstrated to regulate this process 13. Recent studies have demonstrated that the development of several kidney diseases is significantly influenced by exosome-mediated communication among renal tubular epithelial cells, inflammatory cells, and fibroblasts 14-17.

MiRNAs are single-stranded noncoding RNAs that are involved in posttranscriptional gene regulation. Studies have shown that tubular epithelial-derived exosomal miRNAs can promote UIRI-induced renal fibrosis 18. Furthermore, *miR-27a-3p* plays a role in fibrosis development in multiple organs 19, 20, and its expression is significantly downregulated in AKI 21. However, how exosomal *miR-27a-3p* affects renal fibrosis induced by IRI is unknown.

The circadian clock is composed of two parts: a core component in the hypothalamic suprachiasmatic nucleus and an accessory circadian clock in peripheral tissues. As a transcriptional activator, brain and muscle ARNT-like 1 (*BMAL1*) plays important roles in ureteral obstruction-induced renal fibrosis by regulating the transcription of downstream genes 22, 23. Recently, Zhang *et al*. confirmed that *BMAL1* could regulate exosomal miRNAs in adipocytes 24. However, whether *BMAL1* plays a role in renal fibrosis by regulating exosomal miRNAs has not been determined.

Myofibroblasts (defined by alpha-SMA expression) are the major source of the collagen matrix that characterizes tissue fibrosis 25. A recent study revealed that macrophages can directly differentiate into *α-SMA*^+^ myofibroblasts, which then produce a collagen matrix in the injured kidney. This process, known as macrophage-to-myofibroblast transition (MMT), can lead to renal fibrosis 26-28. However, whether MMT is regulated by tubular epithelial-derived exosomes in renal IRI has not been studied.

In this study, we found that *BMAL1* regulated IRI-induced MMT and renal fibrosis by controlling the level of *miR-27a-3p* in tubular epithelial-derived exosomes. Our findings offer a new direction for alleviating or delaying renal fibrosis progression.

## Materials and Methods

### Animals

**Figure 1:**
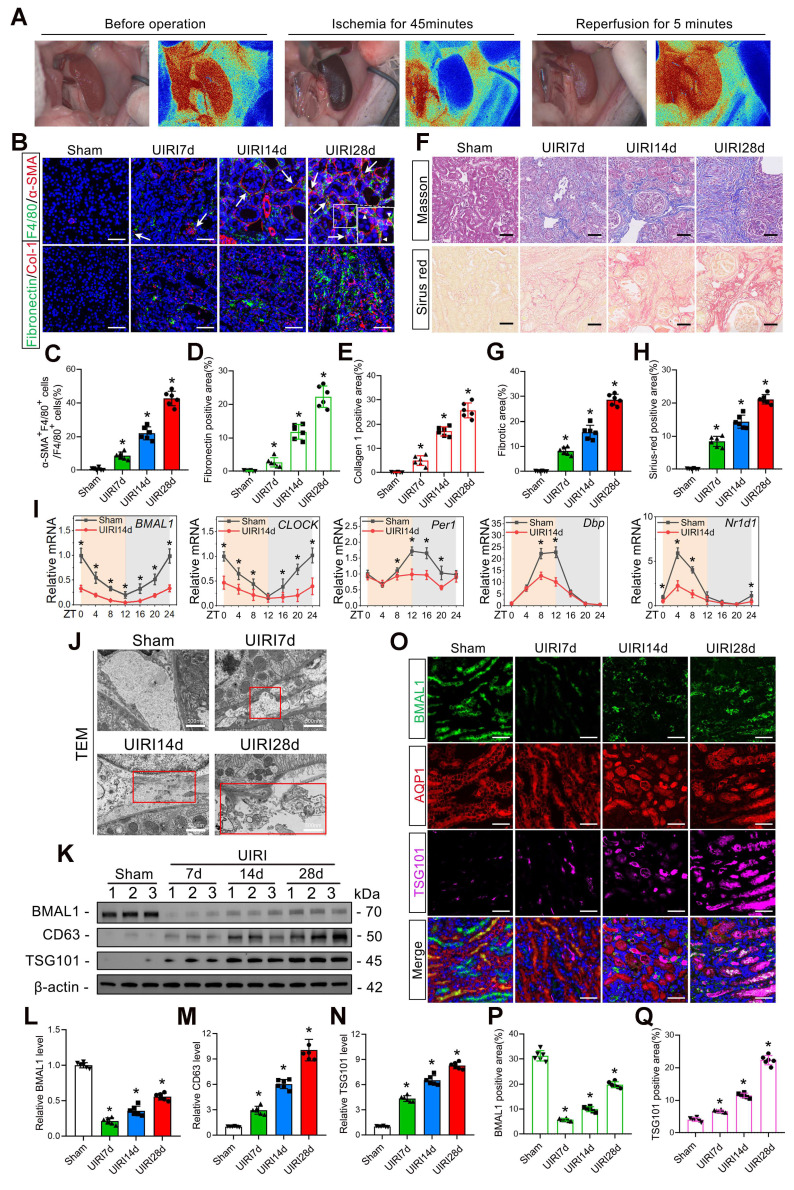
***BMAL1* is downregulated in IRI-induced renal fibrosis models, leading to increased exosome secretion and MMT. A:** A MOORFLPI-2 Laser Speckle Contrast Imager was used to detect renal blood flow during IRI. **B–E:** Representative immunofluorescence staining images **(B)** and quantitative analysis **(C–E)** showing the proportion of MMT cells (*F4/80* and *α-SMA* double-positive cells shown by arrows) and the deposition levels of fibronectin and collagen 1 in the kidney at 7 d, 14 d and 28 d after the UIRI operation. Scale bars = 50 μm. *p < 0.05 versus the sham group. **F–H:** Representative Masson's trichrome and Sirius red staining images **(F)** and quantitative analysis **(G, H)** showing the levels of collagen fiber deposition in the kidneys at 7, 14 and 28 days after the UIRI operation. Scale bars = 50 μm. *p < 0.05 versus the sham group.** I:** qPCR results showing the levels and circadian oscillations of 5 circadian rhythm genes (*BMAL1*, *CLOCK*, *PER1*, *DBP*, and *NR1D1*) in the sham group and UIRI14d group. *p < 0.05 versus the sham group. **J:** Representative TEM images showing extracellular vesicles (as shown in the red box) secreted by renal tubular epithelial cells at 7, 14 and 28 days after the UIRI operation. Scale bars = 500 nm. **K–N:** Representative western blot **(K)** and quantitative analysis **(L–N)** showing the expression levels of *BMAL1*, *CD63* and *TSG101* in the sham, UIRI7d, UIRI14d and UIRI28d groups. *p < 0.05 versus the sham group.** O–Q:** Representative immunofluorescence staining images **(O)** and quantitative analysis **(P, Q)** showing the localization and expression levels of *BMAL1* and *TSG101* in proximal renal tubular epithelial cells in the kidneys of the 4 groups. Scale bars = 50 μm. *p < 0.05 versus the sham group.

Male mice with Rab27a gene deletion (*Rab27a*^-/-^), weighing between 20 and 25 g, were purchased from Cyagen Biosciences (Guangzhou, China). Male C57BL/6 mice, weighing between 20 and 25 g, were obtained from Wuhan University's Center of Experimental Animals (Hubei, China). All the mice used were littermates. Following established protocols 29, a renal unilateral ischemia–reperfusion injury (UIRI) model was established by left renal pedicle clamping with a microvascular clamp followed by reperfusion. Renal blood flow was observed with a MOORFLPI-2 Laser Speckle Contrast Imager (Figure 1A). C57BL/6 mice were divided randomly into four experimental cohorts corresponding to different postoperative time intervals (7, 14, and 28 d following surgery: the sham, UIRI7d, UIRI14d, and UIRI28d groups). The treatment was given according to random group allocation, with only the primary researcher aware of the group allocations. The mice were euthanized at the final time point of the treatment, and the kidneys were collected for subsequent experiments.

To investigate the effects of *BMAL1*, C57BL/6 mice were injected with AAV9 (OBiO Tech, Shanghai, China) carrying the *BMAL1* gene with the KSP-cadherin promoter (AAV9-*BMAL1*) or the control virus (AAV9-*NC*) through the tail vein. After 30 days, the mice were subjected to UIRI.

To determine the function of the exosome contents, exosomes extracted from TCMK-1 cells treated with or without hypoxia–reoxygenation (H/R) were intravenously administered at 1, 4, 7, 10, and 13 days following UIRI (200 μg per mouse per time point). In addition, in accordance with the experimental design, TCMK-1 cells were subjected to different treatments prior to H/R (additional details are presented in the “cell culture and treatment” section).

To examine the function of macrophages in UIRI-induced renal fibrosis, mice were treated with commercially available clodronate liposomes (Clo-Lip) (YEASEN Biotechnology, Shanghai, China) to achieve systemic macrophage depletion. Briefly, 200 μL per mouse of Ctrl-Lip or Clo-Lip was injected through the tail vein 1 day before UIRI and every 3 days after UIRI (1 day before UIRI and 1, 4, 7, 10, and 13 days after UIRI).

**Figure 2:**
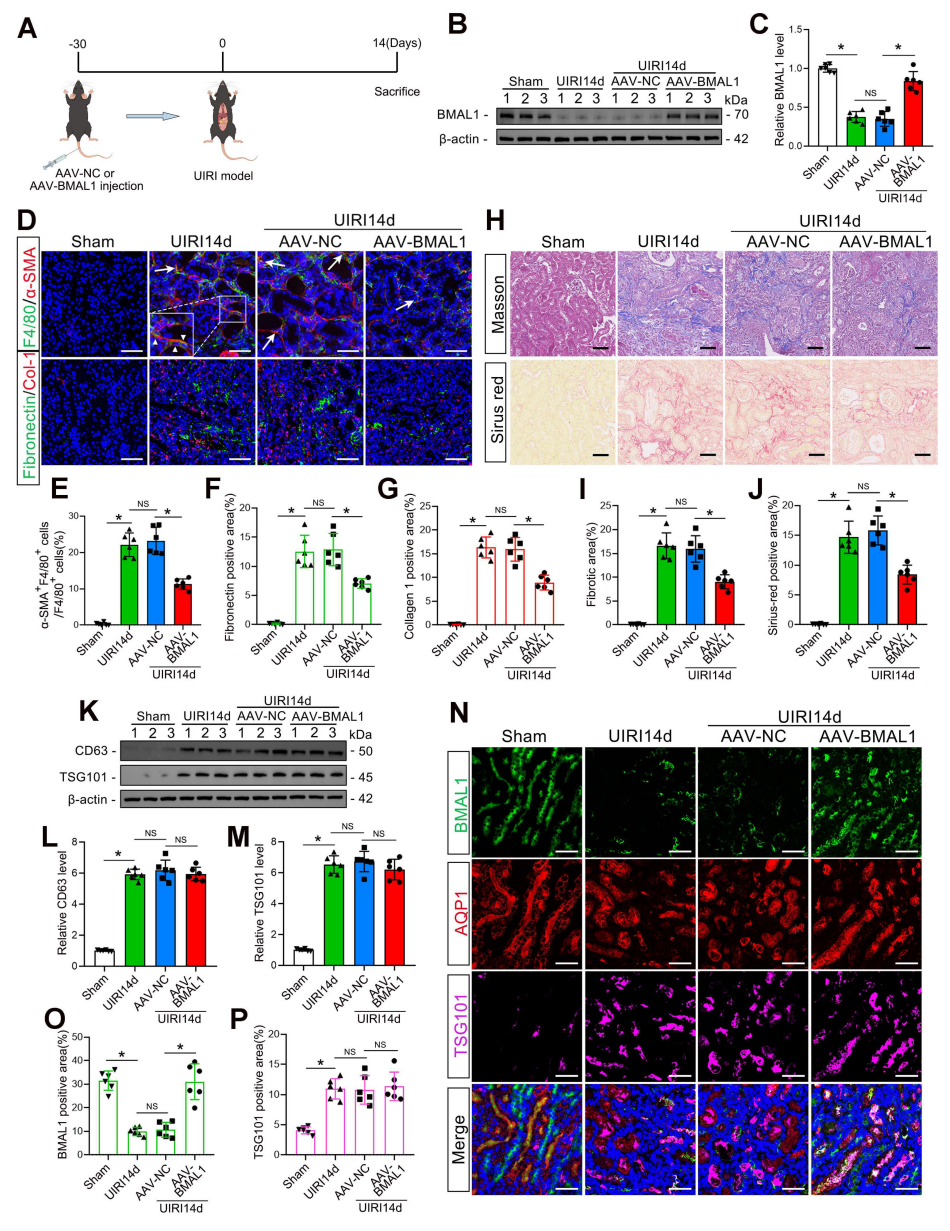
** Overexpression of *BMAL1* decreased renal IRI-induced MMT and renal fibrosis but did not affect the amount of exosomes. A:** Diagram of adenovirus injection and UIRI modeling. **B, C:** Representative western blot** (B)** and quantitative analysis** (C)** showing that adenovirus injection successfully promoted *BMAL1* expression in the kidney. *P < 0.05. NS, not significant. **D–G:** Representative immunofluorescence staining images **(D)** and quantitative analysis **(E–G)** showing that AAV-*BMAL1* treatment inhibited IRI-induced MMT (as shown by the arrows) and fibronectin and collagen 1 deposition. Scale bars = 50 μm. *P < 0.05. NS, not significant. **H–J:** Representative Masson's trichrome and Sirius red staining images **(H)** and quantitative analysis **(I, J)** showing that AAV-*BMAL1* treatment inhibited IRI-induced collagen fiber deposition. Scale bars = 50 μm. *P < 0.05. NS, not significant. **K‒M:** Representative western blot** (K)** and quantitative analysis **(L, M)** showing the levels of *CD63* and *TSG101* in mouse kidneys after AAV-*BMAL1* injection. *P < 0.05. NS, not significant. **N–P:** Representative immunofluorescence staining images **(N)** and quantitative analysis **(O, P)** showing the localization and expression levels of *BMAL1* and *TSG101* in proximal renal tubular epithelial cells after AAV-*BMAL1* injection. Scale bars = 50 μm. *P < 0.05. NS, not significant.

The details of the animal experimental design are shown in Figures 2, 4 and 8. There were 6 mice in each group. Every experimental procedure was authorized by Wuhan University's Animal Experimental Ethics Committee (WDRM 20200308A). All procedures strictly followed the AVMA Guidelines for the Euthanasia of Animals and the Care and Use of Laboratory Animals. The ARRIVE reporting guidelines were used 30.

### Cell culture and treatment

Renal tubular epithelial cells from mice (TCMK-1; ATCC^®^ CCL-139^TM^; Beijing, China) were maintained in MEM/EBSS (HyClone, Cytiva) under standard culture conditions (37 °C, 5% CO_2_). To establish H/R conditions, TCMK-1 cells were initially cultivated for 24 h in medium devoid of exosomes in a three-gas incubator containing 94% N_2_, 5% CO_2_, and 1% O_2_. These cells subsequently underwent reoxygenation for 12 h in an environment containing 21% O_2_, 5% CO_2_, and 74% N_2_.

In accordance with the guidelines provided by the manufacturer, TCMK-1 cells were transfected with siRNAs (si-*NC*, si-*TGFβ1*, si-*Rab27a*, or si-*BMAL1*) using Lipofectamine 2000 (Thermo Fisher, China) to suppress the expression of *TGFβ1*, *Rab27a*, or *BMAL1*.

The lentiviral vector LV-*BMAL1* was used for lentiviral infection to achieve stable overexpression of *BMAL1*, while the empty vector LV-*NC* served as the control group. TCMK-1 cells were transfected with either *miR-27a-3p* mimics or inhibitors according to standard protocols to overexpress and inhibit *miR-27a-3p*, respectively. All of the inhibitors, mimics, lentiviral vectors, and siRNAs were acquired from OBiO Tech (Shanghai, China).

To obtain bone marrow-derived macrophages (BMDMs), researchers collected bone marrow cells from the tibias and femurs of 8-week-old C57BL/6 mice. These cells were then maintained for 7 d in RPMI 1640 culture medium supplemented with 10% FBS and macrophage colony-stimulating factor (M-CSF) at a concentration of 30 ng/mL (R&D Systems, Minneapolis, MN). Lipofectamine 2000 was used to transfect the BMDMs with si-*NC* or si-*TGFBR1* (OBiO Tech, Shanghai, China). For exosome uptake, BMDMs were exposed to exosomes (30 μg protein/mL) that had been purified from TCMK-1 cells for 48 h.

### Exosome isolation

The exosomes were separated by differential centrifugation at 4 °C. In short, the TCMK-1 medium supernatant was collected and centrifuged at 300×g for 10 min, 2000×g for 20 min, and 10,000×g for 30 min to eliminate the cells and debris. The supernatant was ultracentrifuged at 100,000×g for 2 h to isolate the exosomes (OptimaXE-100; Beckman). To eliminate contaminating proteins, the sample was resuspended in phosphate-buffered saline (PBS) and subjected to another ultracentrifugation cycle. The purified exosomes were subsequently resuspended in PBS and quantified for further research.

### Nanoparticle tracking analysis (NTA) of extracellular vesicles

The samples were subjected to NTA to assess the size and quantity of the extracted extracellular vesicles. In short, the samples were first diluted and then calibrated using polystyrene beads. Measurements were subsequently performed using a ZetaView PMX110 nanoparticle analyzer (Particle Metrix, Meerbusch, Germany).

### Transmission electron microscopy (TEM)

Renal tissue samples from mice were initially fixed in 2.5% glutaraldehyde solution, followed by treatment with 3% osmium tetroxide (OsO_4_) for 2 h. After sequential dehydration through ascending ethanol concentrations, the samples were sectioned into ultrathin slices (50–70 nm thickness). Following staining with uranyl acetate and lead citrate, ultrastructural examination was conducted using a Hitachi H7500 transmission electron microscope (Japan).

For extracellular vesicles, the collected samples were first fixed with a PBS solution containing 1% glutaraldehyde and subsequently applied onto specialized grids. After 10 min of uranyl acetate staining, the grids were observed using TEM.

### Labeling and tracking of exosomes

For *in vitro* experiments, TCMK-1-derived exosomes were first collected. The separated exosomes were then subjected to a 10-minute incubation with PKH26 dye (Umibio, China), followed by thorough washing with PBS to eliminate any residual unbound dye. Afterward, the BMDMs were exposed to the labeled exosomes (at a concentration of 3.5×10^7^ particles/mL) for a duration of 48 h.

For animal studies, isolated exosomes were counted and quantified before being incubated with DiR or DiD (Umibio, China) for 30 min. After the excess dye was removed through washing with PBS, the labeled exosomes were administered to the mice through tail vein injection. The biodistribution of these exosomes was then monitored using *in vivo* imaging.

### High-throughput miRNA sequencing

TCMK-1 cells infected with LV-*NC* or LV-*BMAL1* were treated with or without H/R, and exosomes from three groups of cells, namely, LV-*NC*-Exo, H/R-LV-*NC*-Exo and H/R-LV-*BMAL1*-Exo, were subsequently collected and purified. Wayen Biotechnology Company (Shanghai, China) then performed total exosomal RNA extraction (including the small RNA fraction), quality assessment, cDNA library preparation, and HiSeq/MiSeq sequencing.

### Western blot

Following standard experimental protocols, animal and cell samples were lysed for 30 min in ice-cold RIPA lysis solution (Beyotime, China) supplemented with phosphatase inhibitor cocktail (Beyotime, China) and 0.1 mM phenylmethylsulfonyl fluoride (Beyotime, China). Protein quantification was performed using a bicinchoninic acid assay kit (Beyotime, China). The protein samples were separated on 12% or 15% SDS‒PAGE gels before being electrotransferred onto PVDF membranes. These membranes were initially blocked with 5% skim milk, followed by overnight exposure to primary antibodies at 4 °C. After three thorough washing cycles were completed, the membranes were subjected to secondary antibody treatment (either goat-derived anti-mouse IgG or anti-rabbit IgG) for 60 min at room temperature. The protein bands were visualized using a ChemiDoc™ Touch Imaging System (Bio-Rad, USA) and subsequently analyzed with ImageJ software. The complete antibody information is provided in Supplementary Table S1.

### Chromatin immunoprecipitation (ChIP) assay

In summary, the experimental procedure involved sample preparation using a ChIP Assay Kit (Beyotime, China) following cell fixation with 1% formaldehyde. The protein‒DNA complexes were subjected to overnight incubation at 4 °C with specific antibodies. After elution, cross-link reversal, and purification, the isolated DNA was analyzed through both agarose gel electrophoresis and quantitative real-time PCR (qRT‒PCR). The primary antibodies employed in this study included IgG (1:50 dilution; #3900; Cell Signaling Technology) and anti-*BMAL1* (1:50 dilution; #14020; Cell Signaling Technology). The primers used for *BMAL1* binding site detection were as follows: forward, AGCCACTCTTAACCACTGAGTC; reverse, ACCAGGCCTTGGACAGTACTAT.

### qRT‒PCR analysis

Total RNA was isolated from biological specimens, including animal tissues and cultured cells, using TRIzol reagent. RNA was quantified with a NanoDrop (Thermo Fisher, China). To analyze mRNA expression, RNA samples were converted to cDNA using a reverse transcription kit (G3337-50; Servicebio). Gene expression was subsequently quantified through qRT‒PCR with SYBR Green qPCR Master Mix (G3325-01; Servicebio).

For miRNA analysis, exosomal miRNAs were initially purified with the SeraMir Exosome RNA Purification Kit (System Biosciences, USA). Afterward, the Bulge-Loop^TM^ miRNA qRT‒PCR Starter Kit was used for reverse transcription, followed by real-time fluorescence quantitative PCR with the miRNA forward and reverse primers. U6 small nuclear RNA functioned as the normalization control. All PCRs were conducted on a Roche Lightcycler 480II, with the primer sequences detailed in Table S2.

### Luciferase reporter assay

The* wild-type TGFBR1* 3' untranslated region (*WT TGFBR1* 3' UTR) containing the *miR-27a-3p* binding sequence, along with its mutated control (*Mut TGFBR1* 3' UTR), were amplified through polymerase chain reaction. These DNA fragments were subsequently inserted into the pMIR-REPORT plasmid to generate luciferase reporter constructs. BMDMs were transfected with either *miR-27a-3p* mimics or inhibitors alongside the reporter plasmids, and Lipofectamine 2000 was used as the transfection reagent. Following 36 h of incubation, cellular lysates were prepared, and their luciferase activity was quantified using a dual-luciferase detection system (Promega, Madison, WI, USA).

### Flow cytometry

Briefly, BMDMs subjected to various experimental conditions were fixed and permeabilized with a Fixation/Permeabilization Kit (554714; BD Biosciences). These cells were subsequently incubated with anti-*α-SMA* antibodies (NBP2-34522; Novus Biologicals) and anti-*F4/80* antibodies (F0995E; Elabscience). Following staining, the cells were collected and subjected to analysis via flow cytometry (Beckman Coulter Life Sciences, China). With respect to kidney samples from mice, single-cell suspensions were prepared and then exposed to anti-*F4/80* antibodies (11-4801-82; Invitrogen). A LIVE/DEAD™ Fixable Aqua Viability Kit (Cat. No. L34991) was used to distinguish and isolate living cells. The results were analyzed using FlowJo software (v.10.8.1).

### Histology and immunofluorescence staining

Animal and cell samples were prepared according to the usual protocol. Multiple staining techniques, including immunofluorescence, Sirius red, and Masson's trichrome staining, were used, and all the procedures were performed using established methods. TSA fluorescence kits (Servicebio, China) were used for multiplex immunofluorescence staining. Finally, a microscope (Olympus BX51) was used to acquire images. The complete antibody information is provided in Supplementary Table S3.

### Statistical analyses

The experimental data were analyzed using SPSS version 19.0, and the results are presented as the means ± standard. For comparisons involving multiple groups, we applied one-way ANOVA with subsequent Dunnett’s post hoc test. When differences between two groups were compared, Student’s t test was used. Statistical significance was established at a probability threshold of a p value less than 0.05.

## Results

### *BMAL1* is downregulated in IRI-induced renal fibrosis models accompanied by increased exosome secretion and MMT

First, we used immunofluorescence staining for macrophage (*F4/80*) and myofibroblast (*α-SMA*) markers to identify MMT cells at different times after UIRI modeling. The findings demonstrated that the quantity of MMT cells increased significantly with increasing time after UIRI, and the level of MMT was positively proportional to the extracellular matrix (ECM) protein deposition level and renal fibrosis (Figure 1B–H). In addition, macrophage depletion (using Clo-Lip) significantly inhibited IRI-induced MMT and renal fibrosis (Figure S1B–I). These findings suggested that the MMT had a significant effect on the progression of renal IRI-induced fibrosis.

The levels of five circadian rhythm genes (*BMAL1*, *CLOCK*, *PER1*, *DBP*, and *NR1D1*) in the kidneys of the UIRI14d group were then assessed by qPCR. We found that the expression of all five genes decreased and *BMAL1* exhibited the most substantial decrease (Figure 1I). Moreover, we detected significant downregulation of *BMAL1* expression at 6 h after IRI (Figure S1A). Western blotting, immunofluorescence, and TEM were utilized to further examine the function of BMAL1 and exosomes in renal IRI. TEM revealed that the number of extracellular vesicles increased significantly after IRI and was positively correlated with postoperative time (Figure 1J). Western blotting also confirmed that the exosome markers *TSG101* and* CD63* increased with postoperative time (Figure 1K, M, N). Interestingly, *BMAL1* expression was significantly downregulated during IRI, but its expression recovered with increasing postoperative time (Figure 1K, L). We found that this phenomenon occurs primarily in proximal tubule epithelial cells (Figure 1O–Q) by using the proximal tubule epithelial cell marker *AQP1* for localization. Furthermore, the expression of *BMAL1* in the remaining 7 cell types (glomerular mesangial cells, glomerular endothelial cells, glomerular podocytes, macrophages, neutrophils, fibroblasts, and collecting duct cells) in kidneys with IRI is displayed in Figure S2. These results confirmed that IRI promoted MMT and renal fibrosis, decreased *BMAL1* levels and increased exosome secretion in renal tubular epithelial cells.

### Overexpression of *BMAL1* alleviated renal IRI-induced MMT and fibrosis but did not affect the secretion of exosomes

To determine whether *BMAL1* regulates IRI-induced kidney fibrosis, a kidney-specific *BMAL1* overexpression system was used in mice (Figure 2A–C). Subsequently, the MMT and renal fibrosis levels of the mice subjected to UIRI were measured. The findings demonstrated that *BMAL1* overexpression significantly inhibited MMT and ECM protein deposition in UIRI mouse kidneys and ultimately alleviated renal fibrosis (Figure 2D–J). Adenovirus injection alone did not cause the above changes (Figure S3A–C). The association between *BMAL1* and exosomes was further investigated. Surprisingly, the results revealed that upregulation of *BMAL1* did not affect the release of exosomes (Figure 2K–P, Figure S4A–F). These findings led us to speculate that *BMAL1* might regulate MMT and renal fibrosis by regulating the contents of the exosomes.

### Inhibition of exosome release reduces MMT, ECM protein deposition and renal fibrosis

**Figure 3:**
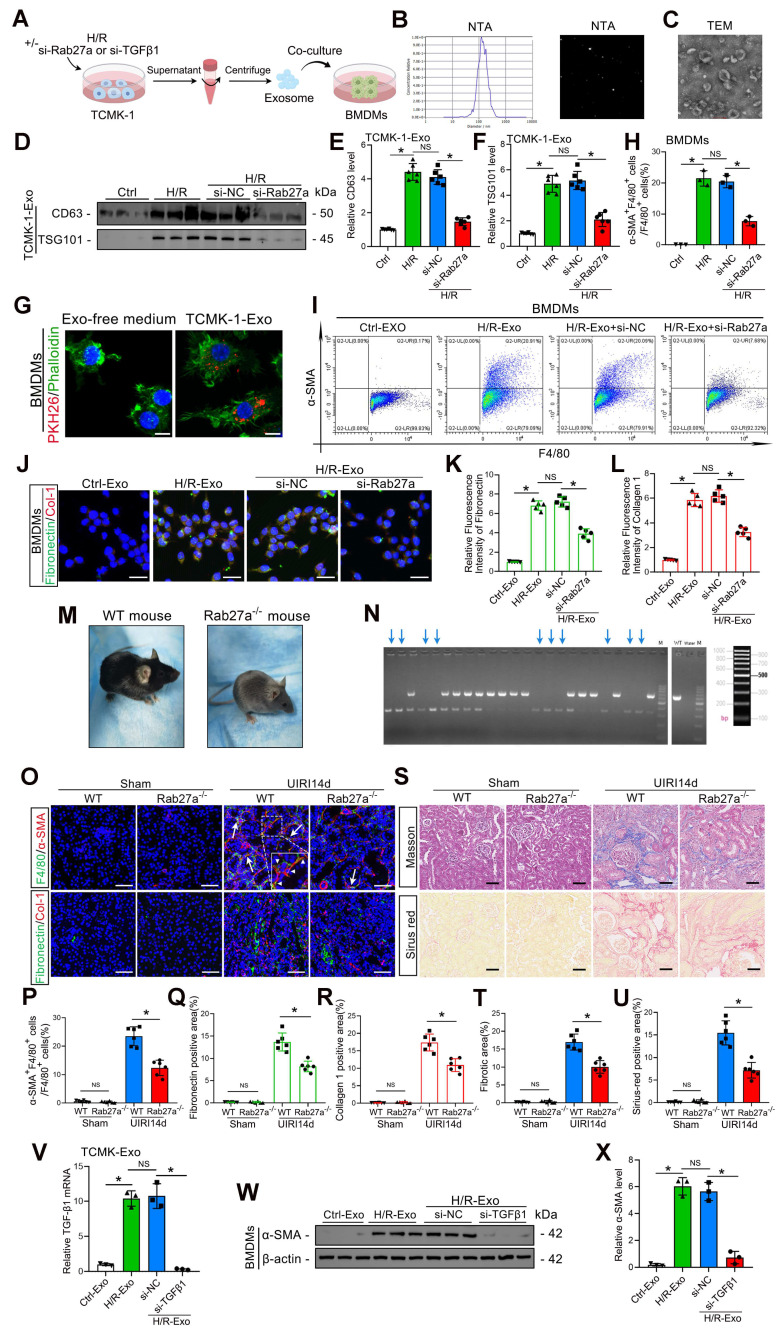
** Inhibition of exosome release can reduce MMT, ECM protein deposition and renal fibrosis. A:** Schematic diagram of the cell experiments. First, TCMK-1 cells were transfected with control siRNA (si-*NC*) or *Rab27a* siRNA (si-*Rab27a*), after which the cells were treated with or without H/R. Then, the exosomes (both Ctrl-Exos and H/R-Exos) were extracted and coincubated with the BMDMs. **B, C:** NTA and TEM images of exosomes from TCMK-1 cells. Scale bars = 100 nm.** D–F:** Representative western blot **(D)** and quantitative analysis **(E, F)** showing the levels of *CD63* and *TSG101* in exosomes derived from TCMK-1 cells after different treatments. *P < 0.05. NS, not significant. **G:** Representative immunofluorescence image of TCMK-1 cell-derived exosomes taken up by BMDMs (green for phalloidin and red for exosomes). Scale bars = 10 μm. **H, I:** Quantitative analysis **(H)** and representative flow cytometry results **(I)** showing *α-SMA* and *F4/80* double-labeled BMDMs. **J–L:** Representative immunofluorescence staining images **(J)** and quantitative analysis **(K, L)** showing fibronectin and collagen 1 expression in BMDMs stimulated with different groups of TCMK-1-derived exosomes. Scale bars = 20 μm. **M:** *WT* mouse and *Rab27a*^-/-^ mouse.** N:** *Rab27a* knockout was confirmed by PCR (target allele: 651 bp; internal control product size: 335 bp). **O-R:** Representative immunofluorescence staining images **(O)** and quantitative analysis **(P–R)** showing the MMT cells (arrows) and deposition levels of fibronectin and collagen 1 in *WT* mice and *Rab27a*^-/-^ mice 14 days after UIRI. Scale bars = 50 μm. *P < 0.05. **S–U:** Representative Masson's trichrome and Sirius red staining images **(S)** and quantitative analysis **(T, U)** showing collagen fiber deposition in *WT* mice and *Rab27a*^-/-^ mice 14 days after UIRI. Scale bars = 50 μm. *P < 0.05. **V:** qPCR results demonstrating the *TGFβ1* mRNA levels in TCMK-1-derived exosomes. **W, X:** Representative western blot **(W)** and quantitative analysis **(X)** results showing *α-SMA* expression in BMDMs. *P < 0.05. NS, not significant.

To clarify whether tubular epithelial-derived exosomes could influence MMT, we transfected TCMK-1 cells with *Rab27a* siRNA to inhibit exosome secretion and subsequently subjected the cells to H/R in exosome-free media. Exosomes were collected and incubated with mouse primary macrophage BMDMs for 48 h (Figure 3A). The majority of the released extracellular vesicles were identified by NTA and TEM as exosomes (based on vesicle diameter), as shown in Figure 3B and C. Furthermore, western blotting results indicated that the secretion of exosomes from TCMK-1 cells was increased by H/R, while the exosome number decreased significantly after the transfection of *Rab27a* siRNA (Figure 3D–F). We then labeled the exosomes with PKH-26, a lipophilic fluorescent dye that is used to trace exosomes, and incubated them for 48 h with BMDMs. Fluorescence microscopy was used to confirm that TCMK-1-derived exosomes were taken up by BMDMs (Figure 3G). Through flow cytometry, the percentage of MMT-positive cells among the BMDMs was determined. The findings demonstrated that treatment with exosomes derived from TCMK-1 cells subjected to H/R significantly promoted MMT (the *α-SMA*^+^*F4/80*^+^ cell ratio exceeded 20%) (Figure 3H, I). Additionally, greater accumulation of ECM proteins (collagen 1 and fibronectin) was detected in MMT cells (Figure 3J–L). Unsurprisingly, these effects were reversed after *Rab27a* siRNA transfection. To further investigate the relationship between exosomes and MMT and renal fibrosis *in vivo*, we constructed *Rab27a* knockout mice (*Rab27a*^-/-^) (Figure 3M, N). As expected, compared with wild-type (WT) mice, *Rab27a*^-/-^ mice exhibited lower MMT and renal fibrosis levels (Figure 3O–U). These results fully confirmed that exosomes from tubular cells can promote MMT and aggravate renal fibrosis during renal IRI.

Furthermore, studies have confirmed that *TGFβ1* mRNA carried by tubuloepithelial-derived exosomes can promote fibroblast activation under H/R conditions 31. To explore whether *TGFβ1* mRNA carried by exosomes is also the key to promoting MMT, we transfected TCMK-1 cells with *TGFβ1* siRNA before H/R treatment and collected exosomes for coincubation with BMDMs (Figure 3A). The qPCR results revealed that the *TGFβ1* mRNA level in TCMK-1-derived exosomes significantly increased after H/R, whereas transfection with *TGFβ1* siRNA decreased the exosomal *TGFβ1* mRNA level (Figure 3V). Moreover, western blot analysis indicated that reducing exosomal *TGFβ1* mRNA expression could inhibit *α-SMA* expression in BMDMs (Figure 3W, X). These results suggest that exosomal *TGFβ1* mRNA is a trigger of MMT.

### *BMAL1* overexpression mitigated tubular epithelial-derived exosome-induced MMT and renal fibrosis

**Figure 4:**
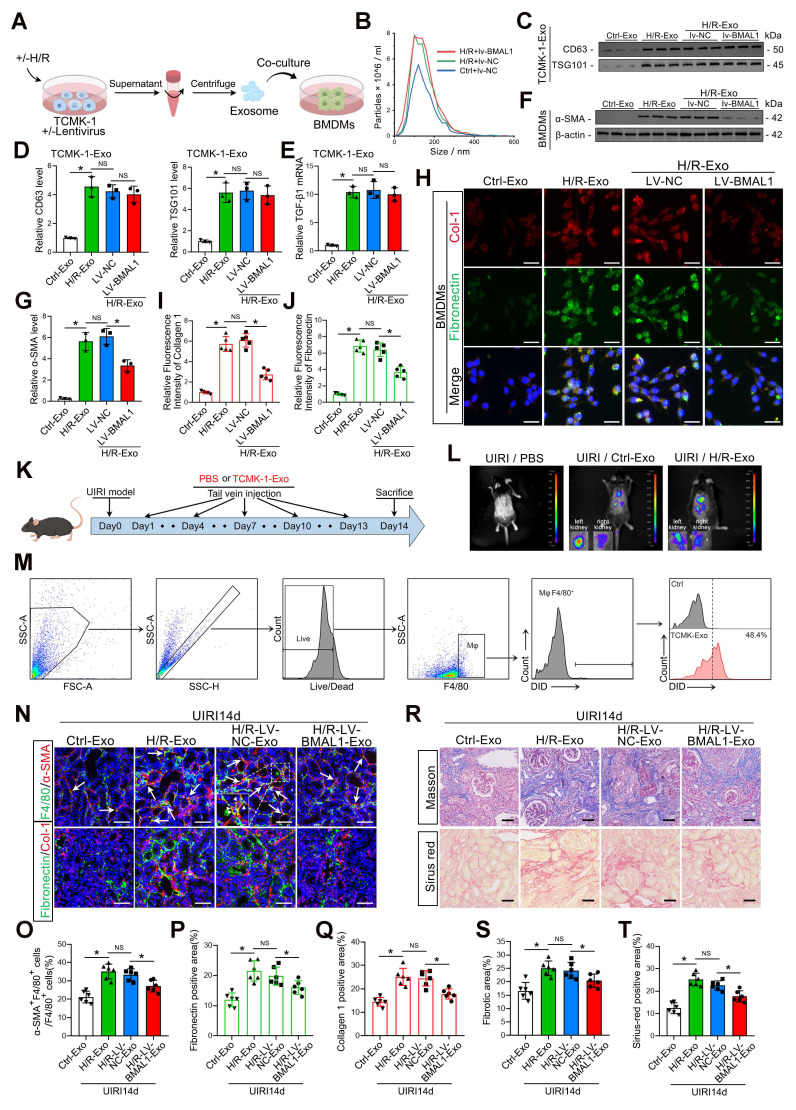
***BMAL1* overexpression mitigated tubular epithelial-derived exosome-induced MMT and renal fibrosis. A:** Schematic diagram of the cell experiments. Lentivirus-*NC* (LV-*NC*) or lentivirus-*BMAL1* (LV-*BMAL1*) was first transfected into TCMK-1 cells, which were subsequently subjected to H/R treatment, after which the exosomes were collected and coincubated with BMDMs.** B:** NTA image of exosomes derived from different groups of TCMK-1 cells. **C, D:** Representative western blot banding **(C)** and quantitative analysis** (D)** of the levels of *CD63* and *TSG101* in the exosomes. *P < 0.05. NS, not significant. **E:** qPCR results showing *TGFβ1* mRNA levels in TCMK-1-derived exosomes after the indicated treatments. *P < 0.05. NS, not significant. **F, G:** Representative western blot **(F)** and quantitative analysis **(G)** showing *α-SMA* expression in BMDMs stimulated with different groups of TCMK-1-derived exosomes. * P < 0.05. NS, not significant.** H–J:** Representative immunofluorescence staining images **(H)** and quantitative analysis** (I, J)** showing collagen 1 and fibronectin expression in BMDMs stimulated with different groups of TCMK-1-derived exosomes. Scale bars = 20 μm. *P < 0.05. NS, not significant. **K:** Exosomes derived from TCMK-1 cells transfected with or without lentivirus (LV-*NC* or LV-*BMAL1*) were collected under H/R conditions and injected into mice through the tail vein at 1 d, 4 d, 7 d, 10 d, and 13 d after UIRI. **L:** Exosomes were first labeled with DIR, after which *in vivo* imaging was used to observe exosome uptake by the kidneys after UIRI. **M:** Exosomes were first labeled with DID, after which flow cytometry was used to observe exosome uptake by macrophages (*F4/80* positive).** N–Q:** Representative immunofluorescence staining images** (N)** and quantitative analysis **(O–Q)** showing the proportion of MMT cells (as shown by the arrows) and the deposition levels of fibronectin and collagen 1 in the kidney after UIRI operation and exosome injection. Scale bars = 50 μm. *p < 0.05. NS, not significant.** R–T:** Representative Masson's trichrome and Sirius red staining images **(R)** and quantitative analysis** (S, T)** showing the levels of collagen fiber deposition in the kidneys after UIRI surgery and exosome injection. Scale bars = 50 μm. *p < 0.05.

Although the function of exosomes in mediating MMT and renal fibrosis has been clarified, the precise connection between *BMAL1* and exosomes in renal IRI remains unclear. Therefore, lentivirus was used to overexpress *BMAL1* in TCMK-1 cells. Exosomes under H/R conditions were subsequently collected and incubated with BMDMs, as shown in Figure 4A. Western blotting and NTA confirmed that prior *BMAL1* overexpression did not affect exosome secretion (Figure 4B–D, Figure S4G). Interestingly, following H/R, the *TGFβ1* mRNA level in exosomes derived from TCMK-1 cells was markedly elevated but was not changed by *BMAL1* overexpression (Figure 4E). However, compared with those in the H/R+LV-*NC* group, the MMT levels in the H/R+LV-*BMAL1* group were significantly lower, and these decreases were accompanied by decreased expression of fibronectin and collagen 1 (Figure 4F–J). These results indicated that *BMAL1* might play a role in renal IRI-induced fibrosis by influencing exosome contents but not by changing *TGFβ1* mRNA levels in exosomes.

To further clarify the role of *BMAL1 in vivo*, we collected four groups of exosomes secreted by TCMK-1 cells in equal amounts and injected them into the tail vein of IRI mice according to the scheme shown in Figure 4K. The exosomes were labeled with DiR, a lipophilic near-infrared fluorescent dye, and large amounts of exosomes taken up by the kidneys were observed via *in vivo* imaging. Interestingly, the uptake efficiency was greater in the left (I/R side) kidney (Figure 4L). We also labeled the exosomes with DiD. The flow cytometry results indicated that macrophages in the IRI kidneys could extensively take up DiD-labeled exosomes (*F4/80* and DiD double-positive cells) (Figure 4M). In addition, exosomes from the H/R-LV-*BMAL1* group significantly reduced MMT and ECM protein deposition in IRI kidneys and ultimately alleviated renal fibrosis (Figure 4N–T, Figure S5A–C).

### *BMAL1* inhibited MMT by upregulating *miR-27a-3p* levels in exosomes from TCMK-1 cells

**Figure 5:**
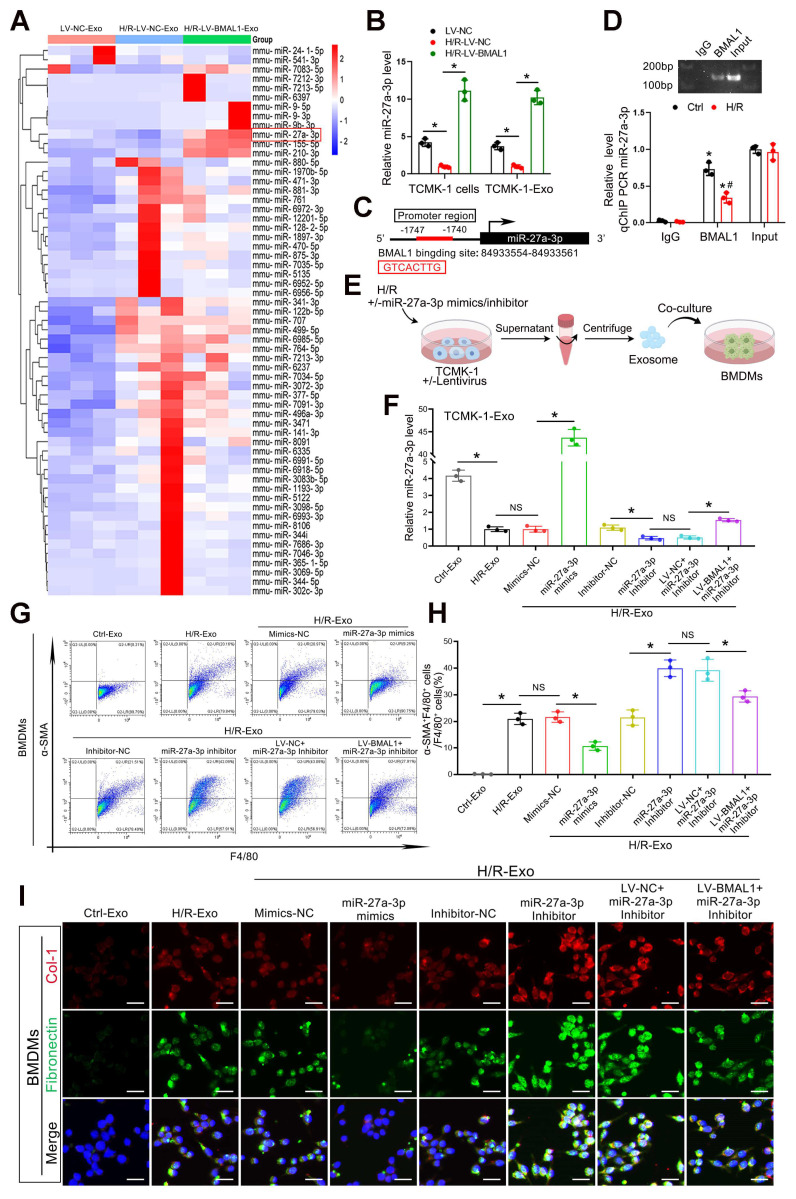
***BMAL1* inhibited MMT by upregulating the level of *miR-27a-3p* in exosomes secreted by TCMK-1 cells. A:** Heatmap of exosomal miRNA sequencing data. Briefly, the cells were divided into LV-*NC*, H/R-LV-*NC*, and H/R-LV-*BMAL1* groups according to the lentivirus used and whether they were subjected to H/R. The exosomes from the three groups were collected for miRNA sequencing. **B:** qPCR results demonstrating the levels of *miR-27a-3p* in TCMK-1 cells and exosomes in the LV-*NC*, H/R-LV-*NC*, and H/R-LV-*BMAL1* groups. *P < 0.05. **C:** JASPAR database showing *BMAL1* binding sites in the *miR-27a-3p* DNA promoter. **D:** Chromatin immunoprecipitation (ChIP) and quantitative ChIP assays were used to determine the recruitment of *BMAL1* to the promoter region of *miR-27a-3p* DNA. All values were initially expressed relative to the immunoglobulin G (IgG) DNA content. *P < 0.05 versus IgG. #P < 0.05. **E:** TCMK-1 cells were first transfected with or without lentivirus (LV-*NC* or LV-*BMAL1*), followed by transfection with mimics-*NC*, *miR-27a-3p* mimics, inhibitor-*NC* or *miR-27a-3p* inhibitor, and finally treated with or without H/R. Exosomes from each group were collected and incubated with BMDMs. **F:** The levels of *miR-27a-3p* in TCMK-1 cell-derived exosomes were measured by qPCR. *P < 0.05. NS, not significant. **G, H:** Representative flow cytometry results **(G)** and quantitative analysis** (H)** showing *α-SMA* and *F4/80* double-labeled BMDMs. *P < 0.05. NS, not significant. **I:** Representative immunofluorescence staining images showing collagen 1 and fibronectin expression in BMDMs stimulated with different groups of TCMK-1-derived exosomes. Scale bars = 20 μm.

As we demonstrated, *BMAL1* might mitigate MMT and renal fibrosis by influencing the contents of tubular epithelial-derived exosomes (not by changing the level of *TGFβ1* mRNA in exosomes). However, the critical content of these exosomes remains unknown. miRNAs are among the primary molecules transported by exosomes, and numerous studies have demonstrated their involvement in renal fibrosis development. Therefore, we collected exosomes from three groups of TCMK-1 cells, namely, LV-*NC*, H/R-LV-*NC* and H/R-LV-*BMAL1*, and performed miRNA sequencing. As shown in Figure 5A, a total of 59 miRNAs were differentially expressed in H/R-treated exosomes and regulated by *BMAL1*. A total of 50 miRNAs whose expression levels were reversed after *BMAL1* was overexpressed were identified. After screening and filtering, we identified *miR-27a-3p* for further study. We verified that H/R treatment significantly reduced exosomal *miR-27a-3p* levels, whereas overexpression of *BMAL1* led to a surprising increase in *miR-27a-3p* levels in both cells and exosomes (Figure 5B). Furthermore, transfection with si-*BMAL1* significantly reduced the *miR-27a-3p* level in both TCMK-1 cells and exosomes, whereas transfection with *miR-27a-3p* mimics significantly reversed these changes (Figure S6C).

According to the transcription factor prediction database (http://jaspar.genereg.net) analysis, a predicted *BMAL1* binding sequence was found in the *miR-27a-3p* promoter region (Figure 5C). The binding of *BMAL1* to the promoter region was subsequently confirmed by chromatin immunoprecipitation (ChIP) assays (Figure 5D). These findings suggested that *BMAL1* directly bound to the *miR-27a-3p* promoter to increase *miR-27a-3p* transcription. *MiR-27a-3p* mimics or inhibitors were transfected into TCMK-1 cells to either overexpress or inhibit exosomal *miR-27a-3p* to validate its function. The effectiveness of the transfection was confirmed by qPCR (Figure 5E, F). Exosomes from eight different groups of TCMK-1 cells were subsequently collected to stimulate BMDMs. Immunofluorescence staining and flow cytometry demonstrated that the MMT, collagen 1 and fibronectin levels of BMDMs decreased significantly when they were stimulated with exosomes derived from TCMK-1 cells treated with *miR-27a-3p* mimics. Similarly, the MMT, collagen 1 and fibronectin levels of BMDMs significantly increased when they were stimulated with exosomes from TCMK-1 cells treated with *miR-27a-3p* inhibitors. However, following *BMAL1* overexpression, the effect of the *miR-27a-3p* inhibitor was markedly reversed (Figure 5G–I, Figure S6A, B). These findings demonstrate that under H/R conditions, *BMAL1* exerts a regulatory effect on MMT by regulating the *miR-27a-3p* level in exosomes from tubular epithelial cells.

### *TGFBR1* is a direct target of exosomal *miR-27a-3p*

**Figure 6:**
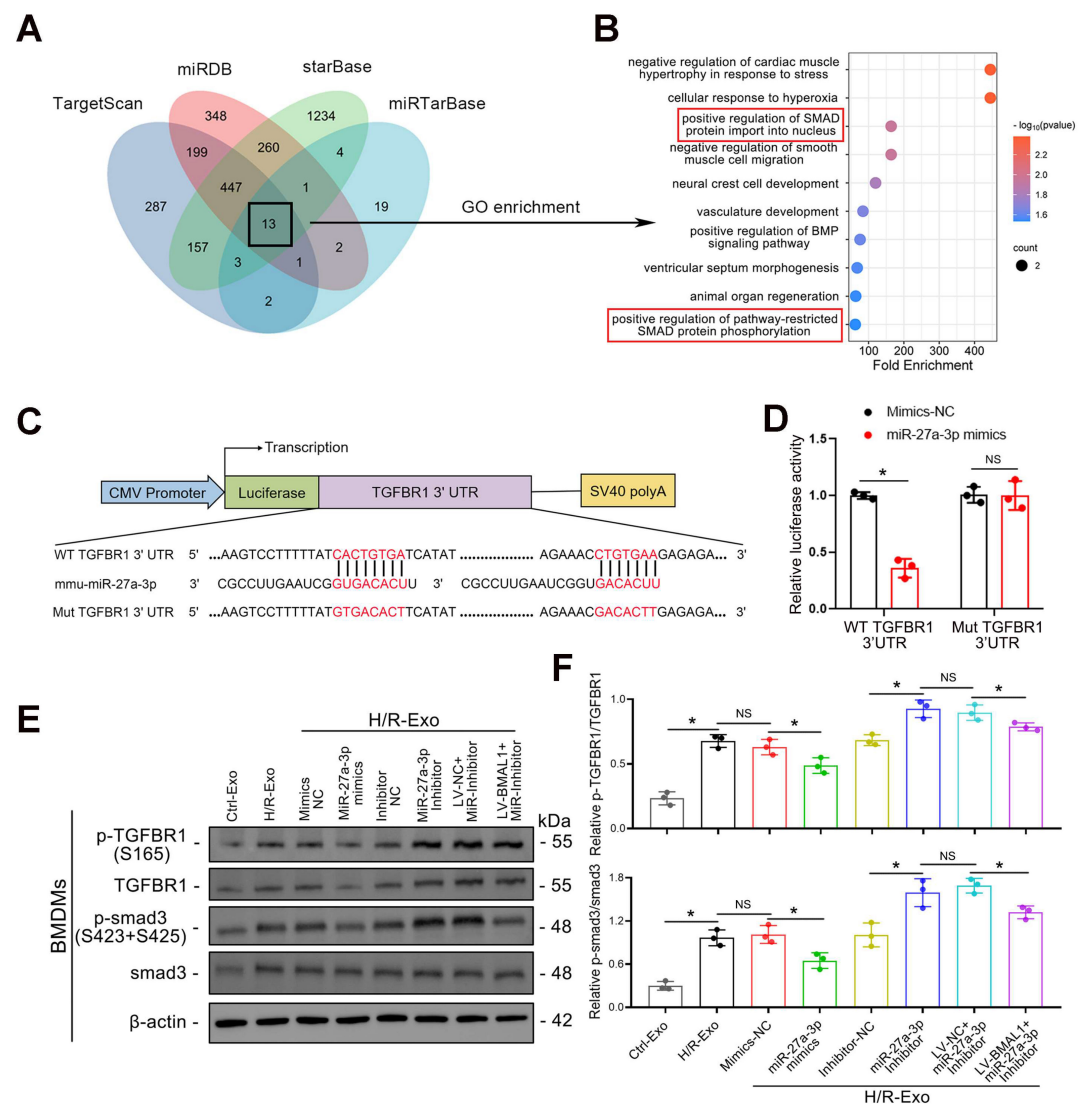
***TGFBR1* is a direct target of exosomal *miR-27a-3p*. A:** Venn diagram showing the intersection of potential target genes of *miR-27a-3p* predicted by four databases: TargetScan, miRDB, starBase and miRTarBase. **B:** The top 10 results of the GO enrichment analysis of the 13 intersecting target genes. **C:** Schematic diagram showing the structure of the luciferase reporter plasmid and the predicted binding sites of the *WT TGFBR1* 3' UTR and mmu-*miR-27a-3p*. **D:** Luciferase activity of cells cotransfected with mimics-*NC* or *miR-27a-3p* mimics and *TGFBR1*-*WT* or *TGFBR1*-*Mut* luciferase reporter plasmids. *P < 0.05. NS, not significant. **E:** Representative western blot showing the levels of *P-TGFBR1*, *TGFBR1*, *P-smad3* and *smad3* in BMDMs stimulated with different groups of TCMK-1-derived exosomes.** F:** Quantitative analysis of *P-TGFBR1/TGFBR1* and *P-smad3/smad3* levels in BMDMs stimulated with different groups of TCMK-1-derived exosomes. *P < 0.05. NS, not significant.

The potential target genes of *miR-27a-3p* were predicted using TargetScan, miRDB, starBase, and miRTarBase. By combining the outputs of the four databases, we were able to identify a total of 13 potential target genes (Figure 6A). GO enrichment analysis revealed that the functions of these 13 genes were enriched in “positive regulation of SMAD protein import into the nucleus” and “positive regulation of pathway-restricted SMAD protein phosphorylation” (Figure 6B). Therefore, we focused our research on *TGFBR1*.

The 3'-untranslated region (3' UTR) of the *TGFBR1* gene was predicted by TargetScan to be the conserved binding site of *miR-27a-3p*, and a luciferase reporter assay verified that *TGFBR1* is a target gene of* miR-27a-3p* (Figure 6C–D). To confirm the regulatory effect of *miR-27a-3p* on *TGFBR1*, the levels of *TGFBR1* and *smad3*, as well as their phosphorylation in BMDMs, were investigated using western blotting. Not surprisingly, *P-TGFBR1/TGFBR1* and* P-smad3/smad3* expression was significantly reduced when BMDMs were stimulated with exosomes secreted by TCMK-1 cells treated with *miR-27a-3p* mimics. In addition, the *P-TGFBR1/TGFBR1* and *P-smad3/smad3* ratios significantly increased when BMDMs were stimulated with exosomes from TCMK-1 cells treated with *miR-27a-3p* inhibitors. However, following the overexpression of *BMAL1*, these changes induced by the *miR-27a-3p* inhibitor were partially reversed (Figure 6E, F). These results suggested that *miR-27a-3p* from renal tubule-derived exosomes directly targeted *TGFBR1* in BMDMs and inhibited *smad3* phosphorylation.

### Renal tubular epithelial cell-derived exosomal *miR-27a-3p* regulated MMT through the *TGFBR1/smad3* pathway *in vitro* during H/R

**Figure 7:**
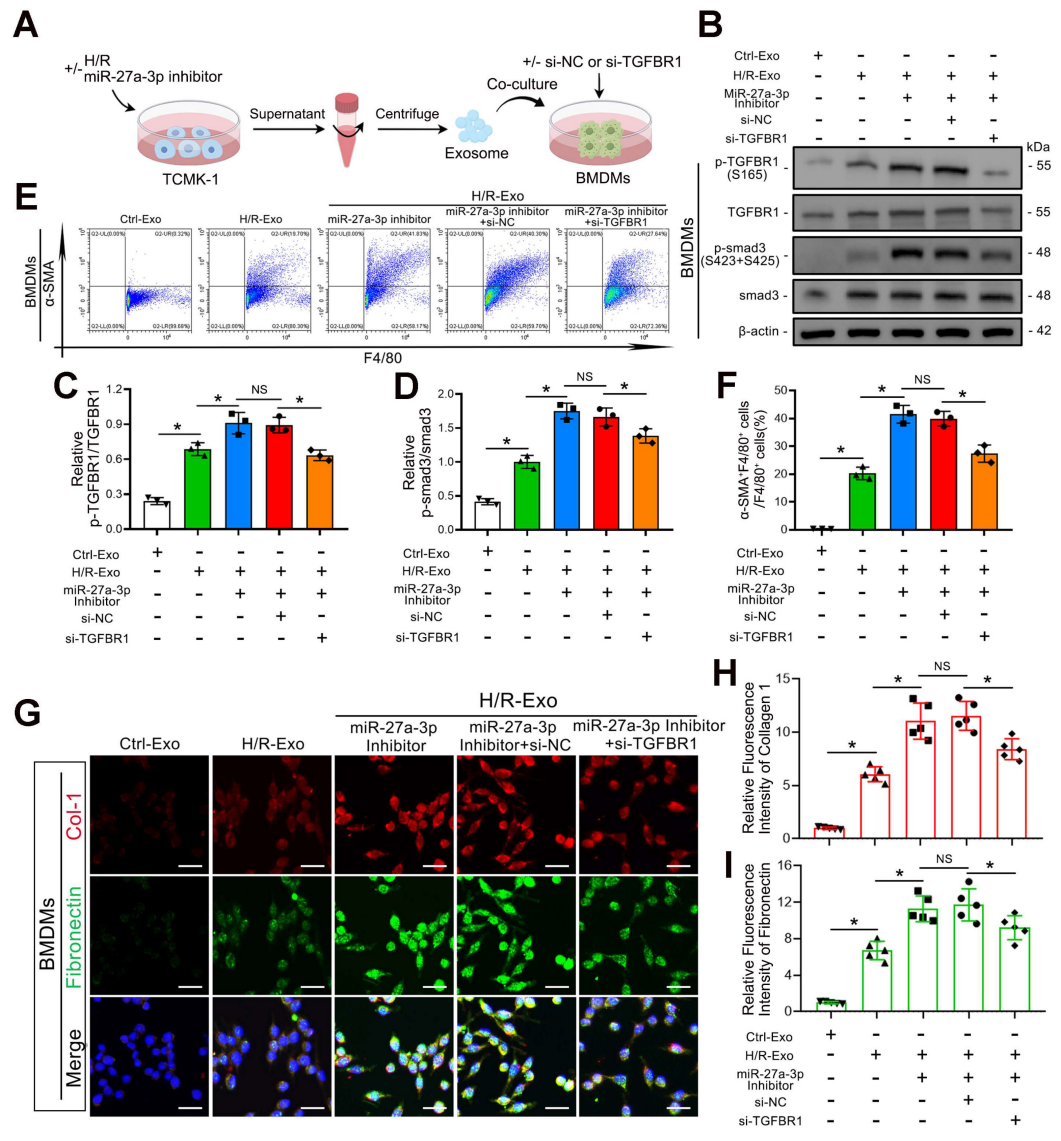
** Renal tubular epithelial cell-derived exosomal *miR-27a-3p* regulated MMT through the *TGFBR1/smad3* pathway *in vitro* during H/R. A:** Schematic diagram of the cell experiments. Briefly, TCMK-1 cells were transfected with or without the *miR-27a-3p* inhibitor and then some groups were subjected to H/R. Moreover, BMDMs were transfected with or without siRNA (si-*NC* or si-*TGFBR1*). Finally, the exosomes derived from TCMK-1 cells were collected and incubated with BMDMs. **B:** Representative western blot showing the expression of *P-TGFBR1*, *TGFBR1*, *P-smad3* and *smad3* in BMDMs after different treatments. **C, D:** Quantitative analysis of *P-TGFBR1/TGFBR1* and *P-smad3/smad3* levels in BMDMs after different treatments. *P < 0.05. NS, not significant. **E, F:** Representative flow cytometry results **(E)** and quantitative analysis **(F)** showing *α-SMA* and *F4/80* double-labeled BMDMs. *P < 0.05. NS, not significant. **G–I:** Representative immunofluorescence staining images **(G)** and quantitative analysis **(H, I)** showing collagen 1 and fibronectin expression in BMDMs after different treatments. Scale bars = 20 μm. *P < 0.05. NS, not significant.

To further confirm whether *TGFBR1* is the main downstream pathway of exosomal *miR-27a-3p*-mediated MMT, we transfected BMDMs with *TGFBR1* siRNA prior to applying TCMK-1 cell-derived exosomes (Figure 7A). Western blot analysis confirmed the silencing efficiency of the *TGFBR1* siRNA. In addition, *TGFBR1* siRNA transfection significantly reversed the increase in *P-TGFBR1/TGFBR1* and *P-smad3/smad3* induced by the exosomes from TCMK-1 cells treated with the *miR-27a-3p* inhibitor (Figure 7B–D). Not surprisingly, the flow cytometry results also revealed that the MMT induced by the exosomes from TCMK-1 cells treated with the *miR-27a-3p* inhibitor was reversed after *TGFBR1* inhibition (Figure 7E, F). Immunofluorescence staining also revealed that the expression of collagen 1 and fibronectin decreased along with exosome-mediated MMT inhibition by *TGFBR1* siRNA (Figure 7G–I). These results confirmed that under H/R conditions, the inhibition of MMT by *miR-27a-3p* from renal tubular epithelial-derived exosomes was achieved through the targeting of *TGFBR1*.

### An increase in renal tubular epithelial cell-derived exosomal *miR-27a-3p* inhibited IRI-induced MMT, ECM protein deposition and renal fibrosis

**Figure 8:**
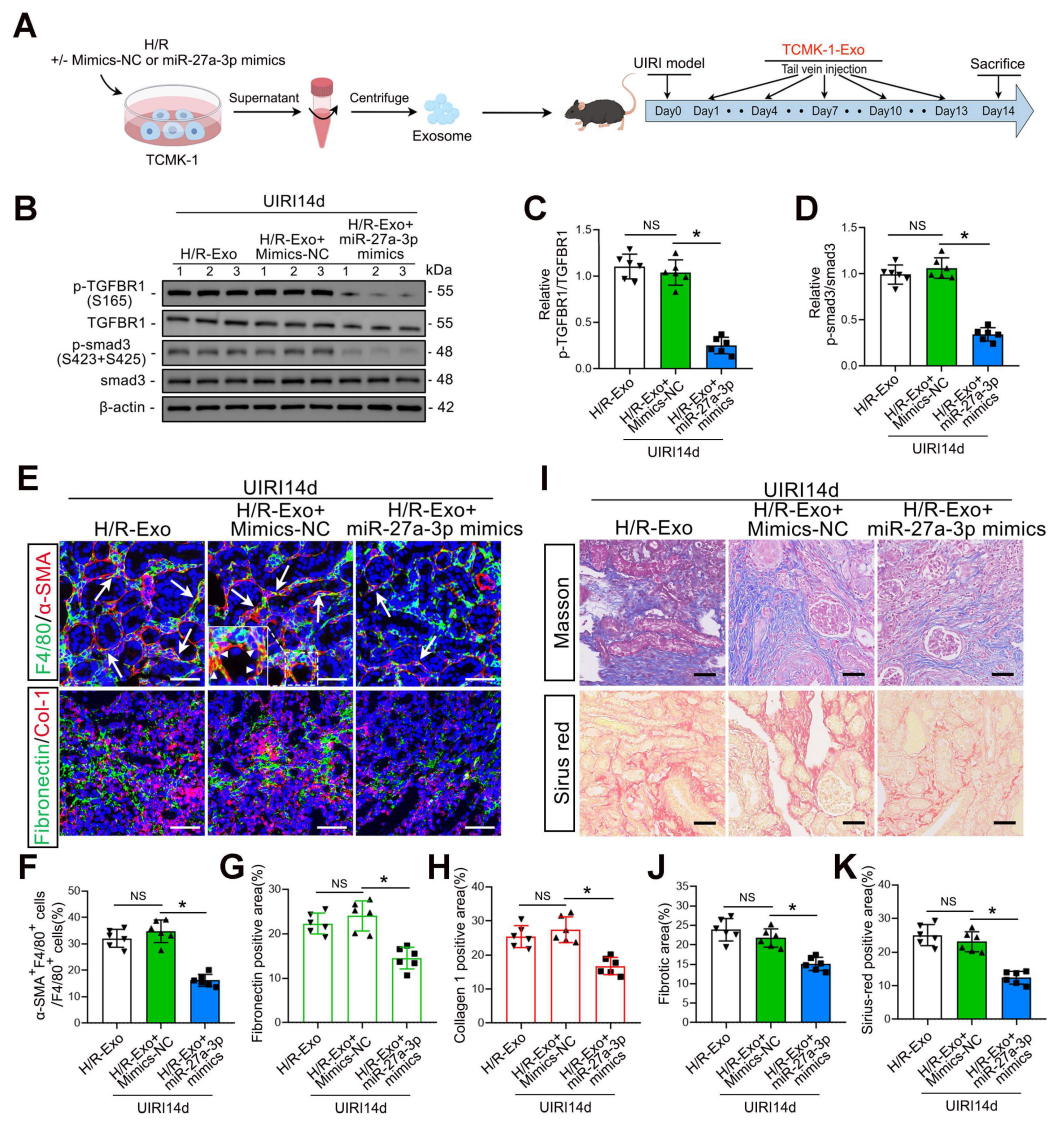
** An increase in renal tubular epithelial cell-derived exosomal *miR-27a-3p* inhibited IRI-induced MMT, ECM protein deposition and renal fibrosis. A:** Schematic diagram of the animal experiments. Briefly, TCMK-1 cells were transfected with mimics-*NC* or *miR-27a-3p* mimics or not, followed by H/R treatment. Exosomes derived from TCMK-1 cells were collected and injected into the tail vein of mice after the UIRI operation. **B:** Representative western blot showing the expression of *P-TGFBR1*, *TGFBR1*, *P-smad3* and *smad3* in the kidneys of different groups. **C, D:** Quantitative analysis of the *P-TGFBR1/TGFBR1* and *P-smad3/smad3* ratios in the kidneys of the different groups. *P < 0.05. NS, not significant. **E–H:** Representative immunofluorescence staining images **(E)** and quantitative analysis **(F–H)** showing the proportion of MMT cells (as shown by the arrows) and the deposition levels of fibronectin and collagen 1 in the kidneys after the UIRI operation and exosome injection. Scale bars = 50 μm. *p < 0.05. NS, not significant. **I–K:** Representative Masson's trichrome and Sirius red staining images** (I)** and quantitative analysis **(J, K)** showing the levels of collagen fiber deposition in the kidneys after UIRI surgery and exosome injection. Scale bars = 50 μm. *p < 0.05. NS, not significant.

As shown in Figure 8A, we injected H/R-Exos into UIRI14d mice to investigate the function of exosomal *miR-27a-3p* from TCMK-1 cells *in vivo*. The results confirmed that the injection of exosomes from TCMK-1 cells treated with *miR-27a-3p* mimics decreased *P-TGFBR1/TGFBR1* and *P-smad3/smad3* levels (Figure 8B–D). Histological staining further confirmed that the injection of exosomes from TCMK-1 cells treated with *miR-27a-3p* mimics significantly inhibited renal MMT and ECM protein deposition, thus alleviating renal fibrosis (Figure 8E–K).

## Discussion

Renal tubular epithelial cells are susceptible to harm from toxic substances, hydronephrosis, and ischemia 32-35. In AKI, extensive and complex crosstalk between renal tubular epithelial cells and stromal cells (including endothelial cells, fibroblasts, and immune cells) plays a significant role in the progression of injury and kidney recovery 36-39. In this study, we found that the number of exosomes released from stressed renal tubular epithelial cells increased significantly during IRI. These morbid exosomes can promote MMT, which directly leads to renal fibrosis, and we confirmed that *BMAL1* could mitigate this exosome-mediated MMT and fibrosis. We further revealed that *BMAL1* inhibited MMT by upregulating exosomal *miR-27a-3p,* which could impair *TGFβ1*-mediated *TGFBR1/smad3* pathway activation. Our findings offer new insights into how *BMAL1* regulates exosome-mediated epithelial and macrophage communication in renal IRI-induced MMT and fibrosis.

*BMAL1* is among the core clock genes that participate in the progression of multiple kidney diseases 22, 40, 41. However, reports on how *BMAL1* regulates renal IRI-induced fibrosis are lacking. Our study demonstrated that restoring renal IRI-induced *BMAL1* depletion inhibited macrophage infiltration and ECM protein deposition in the kidney. These findings are in line with our earlier research showing that *BMAL1* inhibits ureteral obstruction-induced inflammatory cell infiltration and renal fibrosis 22. However, previous studies have focused only on the correlation between *BMAL1* expression and macrophage infiltration, and the possible underlying mechanisms have not been fully elucidated. MMT is a newly identified myofibroblast source that results from abnormal *TGFβ/smad3* pathway activation and has been shown to be directly involved in renal fibrosis. For the first time, our research showed that increased *BMAL1* expression in tubular epithelial cells can prevent renal fibrosis by inhibiting exosome-mediated MMT.

**Figure 9:**
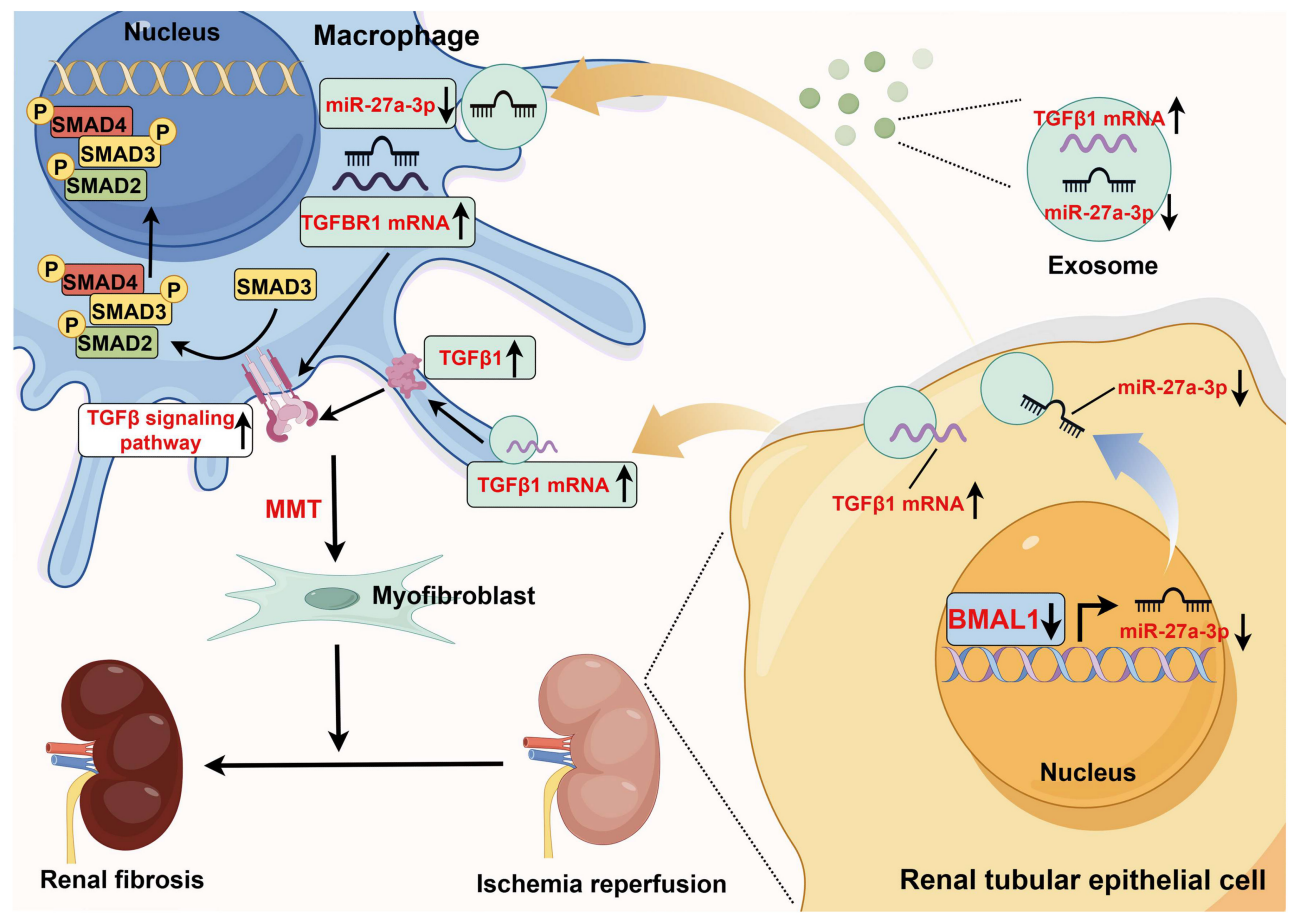
** Schematic diagram of the mechanism of action of *BMAL1* during IRI**. During IRI, the *BMAL1* level in renal tubular epithelial cells is significantly downregulated. Subsequently, by affecting transcription, *BMAL1* directly reduces the level of *miR-27a-3p* in exosomes. Under IRI conditions, the increase in *TGFβ1* mRNA expression and decrease in *miR-27a-3p* expression in exosomes synergistically promote the abnormal activation of the *TGFBR1/smad3* signaling pathway in macrophages, which leads to MMT and renal fibrosis (the figure was created by using Figdraw).

Exosomes are bilayer vesicles that range in diameter from 30 to 150 nm. Their contents are abundant in proteins and nucleic acids, including circular RNAs, lncRNAs, and miRNAs 7-10. Numerous studies have shown that exosome-mediated cellular communication plays a significant role in multifactorial renal fibrosis 14, 42, 43. Our study confirmed that the inhibitory effect of *BMAL1* on MMT was also mediated through exosomes. Multiple experiments revealed that H/R-induced TCMK-1-derived exosomes can be taken up directly by macrophages, promoting MMT and inducing collagen matrix production. However, after *BMAL1* was overexpressed in renal tubular epithelial cells, this phenomenon was significantly reversed. We also found that the *TGFβ1* mRNA level in exosomes was significantly increased, which appears to be the “trigger point” for inducing MMT (Figure 9). As expected, the silencing of *TGFβ1* mRNA in exosomes also inhibited MMT. These findings are similar to those of Borges FT *et al*., who reported that *TGFβ1* mRNA from tubular epithelial-derived exosomes can activate fibroblasts and induce renal fibrosis 31. This might also explain why *Rab27a*^-/-^ mice exhibit lower MMT and fibrosis after UIRI surgery. Unexpectedly, our study revealed that overexpression of *BMAL1* did not affect exosome secretion or exosomal *TGFβ1* mRNA levels. However, prior research has demonstrated that *BMAL1* can promote exosome release by human colorectal cancer 44. We suspect that this difference may be caused by different *BMAL1* functions in different organs and models. These findings suggested that *BMAL1* seems to inhibit MMT by altering exosome contents such as those of *miR-27a-3p,* which could affect the *TGFBR1/smad3* pathway, thus influencing fibrosis rather than directly decreasing exosomal *TGFβ1* mRNA levels.

Previous studies have shown that *BMAL1*, as a transcriptional activator, can promote the transcription of downstream genes, including miRNAs 45. Our study found that *BMAL1* expression was significantly downregulated after H/R stimulation, and this change was accompanied by the downregulation of *miR-27a-3p* expression in both TCMK-1- and TCMK-1-derived exosomes. Using ChIP and qPCR, we verified that *BMAL1* could bind to the *miR-27a-3p* promoter region to increase the transcription of *miR-27a-3p*. Furthermore, there was an inverse relationship between exosomal *miR-27a-3p* levels and exosome-mediated MMT. By upregulating exosomal *miR-27a-3p* expression, *BMAL1* reverses this process. These results reveal that the decrease in exosomal *miR-27a-3p* regulated by *BMAL1* and the increase in exosomal* TGFβ1* mRNA jointly promote MMT during H/R (Figure 9). Similar to our results, JI *et al*. reported that delivery of the *miRNA-23a/27a/26a* cluster by exosomes from satellite cells reduces renal tubulointerstitial fibrosis in mice with diabetic nephropathy 46.

Abnormal activation of the *TGFβ/smad3* pathway is believed to be a central factor in MMT. In brief, when *TGFβ* signaling is activated, the *TGFβ* receptors *TGFBR2* and *TGFBR1* are phosphorylated and activated sequentially. Subsequently, phosphorylated *TGFBR1* recruits and phosphorylates *smad3* to promote MMT. We predicted the target genes of *miR-27a-3p* through four databases. GO enrichment analysis revealed that the functions of these genes were enriched in “positive regulation of SMAD protein import into the nucleus” and “positive regulation of pathway-restricted SMAD protein phosphorylation”. In addition, luciferase reporter assays and PCR confirmed that *miR-27a-3p* can bind to the 3' UTR of *TGFBR1* to inhibit *TGFBR1* expression, directly leading to disruption of the *TGFβ* signaling pathway. In addition, MMT expression was significantly inhibited when *TGFBR1* expression was suppressed in BMDMs by si-*TGFBR1*, further confirming that the *TGFBR1/smad3* pathway is the main downstream pathway through which exosomal *miR-27a-3p* regulates MMT.

Notably, prior to our study, the exosomal protein *OPN* derived from tubular cells was shown to be essential for the activation of fibroblasts 43. In addition, Liu *et al*. reported that hepatic stellate cell activation and cholestatic liver fibrosis were enhanced by cholangiocyte-derived exosomal lncRNA H19 47. Our study did not investigate whether other components in exosomes are also involved in *BMAL1*-mediated regulation of MMT. However, this does not affect the importance of our study. Previous studies focused only on the correlation between *BMAL1* and renal fibrosis but did not thoroughly clarify how *BMAL1* inhibits renal fibrosis by affecting exosome-mediated renal tubular epithelial cell and macrophage communication. In addition, for the first time, our study identified the inhibitory effect of *BMAL1* on MMT.

In conclusion, IRI-induced long-term renal fibrosis is significantly influenced by *BMAL1* expression in renal tubular epithelial cells. *BMAL1* inhibits MMT via the *TGFBR1/smad3* pathway by influencing the delivery of exosomal *miR-27a-3p*. These results introduce a new mechanism to explain how *BMAL1* inhibits MMT through exosome-mediated cellular communication in a renal IRI model, providing a new direction for alleviating or delaying renal fibrosis progression.

## Conclusions

One common pathological alteration that occurs as AKI progresses to CKD is renal fibrosis. Accordingly, how to delay and alleviate fibrosis has been the focus of CKD-related research. Our study confirmed that exosomes from H/R-treated tubular epithelial cells further exacerbated MMT and renal fibrosis in an IRI model, whereas *BMAL1* overexpression or *Rab27a* knockout significantly attenuated IRI-induced MMT and fibrosis progression. Notably, tubular-specific overexpression of *BMAL1*, elevation of exosomal *miR-27a-3p* levels, or inhibition of exosome secretion via si-*Rab27a* significantly attenuated the progression of both MMT and fibrosis. Mechanistic investigations revealed that *BMAL1* directly binds to the *miR-27a-3p* promoter region and enhances its transcription. Exosomal *miR-27a-3p* subsequently targets *TGFBR1* mRNA in macrophages, thereby suppressing the *TGFBR1/smad3* signaling pathway and ultimately attenuating MMT and renal fibrosis. These results introduce a new mechanism to explain how *BMAL1* inhibits MMT through exosome-mediated cellular communication in a renal IRI model, providing a new direction for alleviating or delaying renal fibrosis progression.

## Supplementary Material

Supplementary figures and tables.

Supplementary data.

## Data Availability

The exosomal miRNA sequencing data that support the conclusions of this study are publicly accessible in the repository [GEO: GSE261069] at [https://www.ncbi.nlm.nih.gov/geo/query/acc.cgi?acc = GSE261069]. The remaining data are available within the article and supplementary information.

## Abbreviations

IRI: ischemia‒reperfusion injury
MMT: macrophage-to-myofibroblast transition
AKI: acute kidney injury
H/R: hypoxia–reoxygenation
CKD: chronic kidney disease
miRNAs: microRNAs
lncRNAs: long noncoding RNAs
*BMAL1*: brain and muscle ARNT-like 1
UIRI: unilateral ischemia‒reperfusion injury
Clo-Lip: clodronate liposomes
BMDMs: bone marrow-derived macrophages
NTA: nanoparticle tracking analysis
M-CSF: macrophage colony-stimulating factor
TEM: transmission electron microscopy
ChIP: chromatin immunoprecipitation
qRT–PCR: quantitative real-time PCR
ECM: extracellular matrix
WT: wild type
