# Supplementary material for: BMAL1 regulates tubular epithelial-derived exosomal miR-27a-3p to inhibit macrophage–myofibroblast transition and alleviate ischemia/reperfusion-induced renal fibrosis
Source: Theranostics. 2026 May 18;16(12):6988–7006. doi: 10.7150/thno.127538 (PMC13232619; doi:10.7150/thno.127538)
Supplement: Supplementary file 1 — Supplementary figures and tables. [file thnov16p6988s1.pdf]

Table S1. Details of the antibodies.

Antibodies	Company	Catalog number	Dilutions for WB
BMAL1	Cell Signaling Technology	#14020	1:1000
CD63	Santa Cruz Biotechnology	sc-5275	1:1000
TSG101	Santa Cruz Biotechnology	sc-7964	1:1000
α-SMA	Proteintech	14395-1-AP	1:3000
Fibronectin	Proteintech	15613-1-AP	1:4000
Phospho-TGFBR1	Absin	abs139909	1:2000
TGFBR1	Santa Cruz Biotechnology	sc-101574	1:1000
Phospho-Smad3	Abcam	Ab52903	1:2000
Smad3	Abcam	Ab40854	1:4000
β-actin	Proteintech	66009-1-Ig	1:20000

Table S2. Primer sequence.

Gene product	Nucleotide sequence (5'→ 3')
BMAL1- forward	GGACTTCGCCTCTACCTGTT
BMAL1- reverse	GCTGTCGCCCTCTGATCTAC
CLOCK- forward	TGGTGACTGCCTATCCTACCTTCG
CLOCK- reverse	TGCTGCTGCTGCTGCTGTTG
Per1- forward	CCTGGGCTCTGGGTCTGGTTC
Per1- reverse	TTGCTTGTATGGCTGCTCTGACTG
Dbp- forward	GCTGCTTGACATCTAGGGACACAC
Dbp- reverse	GGAATGCTTGACAGGGCGAGATC
Nr1d1- forward	CTTCCTCCTACCCGCCTACCTG
Nr1d1- reverse	TGTTGCCTTGCCGTAGACTGTTG
TGFβ1- forward	ACCGCAACAACGCCATCTATGAG
TGFβ1- reverse	GGCACTGCTTCCCGAATGTCTG
Actin- forward	GTGACGTTGACATCCGTAAAGA
Actin- reverse	GCCGGACTCATCGTACTCC
RT Primer	GTCGTATCCAGTGCAGGGTCCGAGGTATTCGCACTGGATACGACGCGGAA

miR-27a-3p- forward	AATCGGCGTTCACAGTGGCTAA
miR-27a-3p- reverse	ATCCAGTGCAGGGTCCGAGG
U6- forward	CTCGCTTCGGCAGCACA
U6- reverse	AACGCTTCACGAATTTGCGT

Table S3. Details of the antibodies.

Antibodies	Company	Catalog number	Dilutions for WB
BMAL1	Abcam	ab228594	1:200
CD63	Santa Cruz Biotechnology	sc-5275	1:50
TSG101	Santa Cruz Biotechnology	sc-7964	1:50
α-SMA	Cell Signaling Technology	#19245	1:400
Fibronectin	Proteintech	15613-1-AP	1:200
collagen 1	Proteintech	14695-1-AP	1:200
AQP1	Abcam	ab168387	1:50
AQP2	Abcam	ab199975	1:100
F4/80	Abcam	ab6640	1:40
Vimentin	Cell Signaling Technology	#5741	1:300
CD31	Cell Signaling Technology	#15585	1:300
WT1	Abcam	ab89901	1:100
Ly6g	Cell Signaling Technology	#87048	1:200

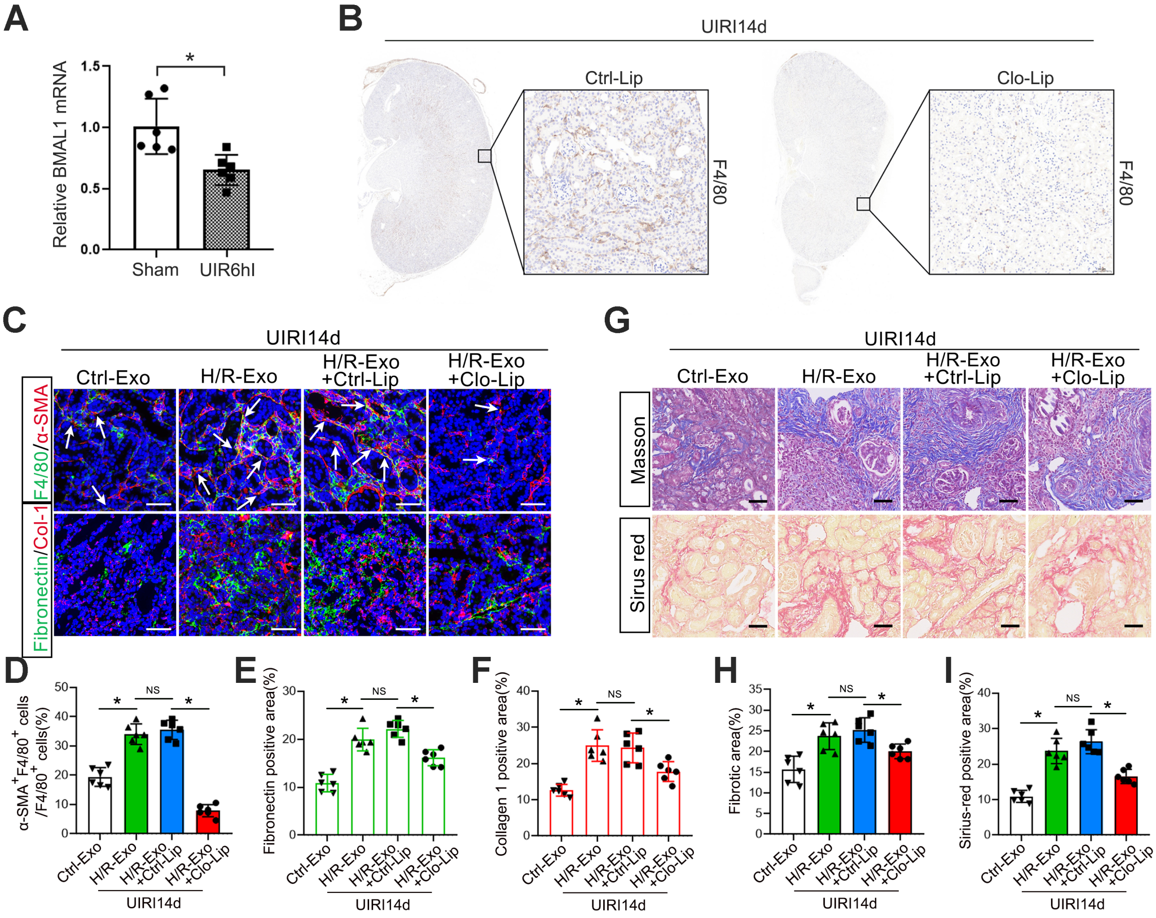

Figure S1. Macrophage depletion mitigated renal fibrosis induced by IRI. A: qPCR results showed the levels of *BMAL1* in Sham and UIR6hI group. **B:** Representative Immunohistochemical staining of *F4/80* showed the levels of macrophage infiltration in the kidneys with Ctrl-Lip or Clo-Lip treatment. Scale bars = 50 μm. **C-F:** Representative immunofluorescence staining images **(C)** and quantitative analysis **(D-F)** showed the proportion of MMT cells (as shown by the arrows) and the deposition levels of fibronectin and collagen 1 in the kidneys of UIRI14d group after exosome injection and with Ctrl-Lip or Clo-Lip treatment. Scale bars = 50 μm. *p < 0.05. NS, not significant. **G-I:** Representative Masson's trichrome and Sirius red staining images **(G)** and quantitative analysis **(H, I)** showed the levels of collagen fibre deposition in the kidneys of UIRI14d group after exosome injection and with Ctrl-Lip or Clo-Lip treatment. Scale bars = 50 μm. *p < 0.05. NS, not significant.

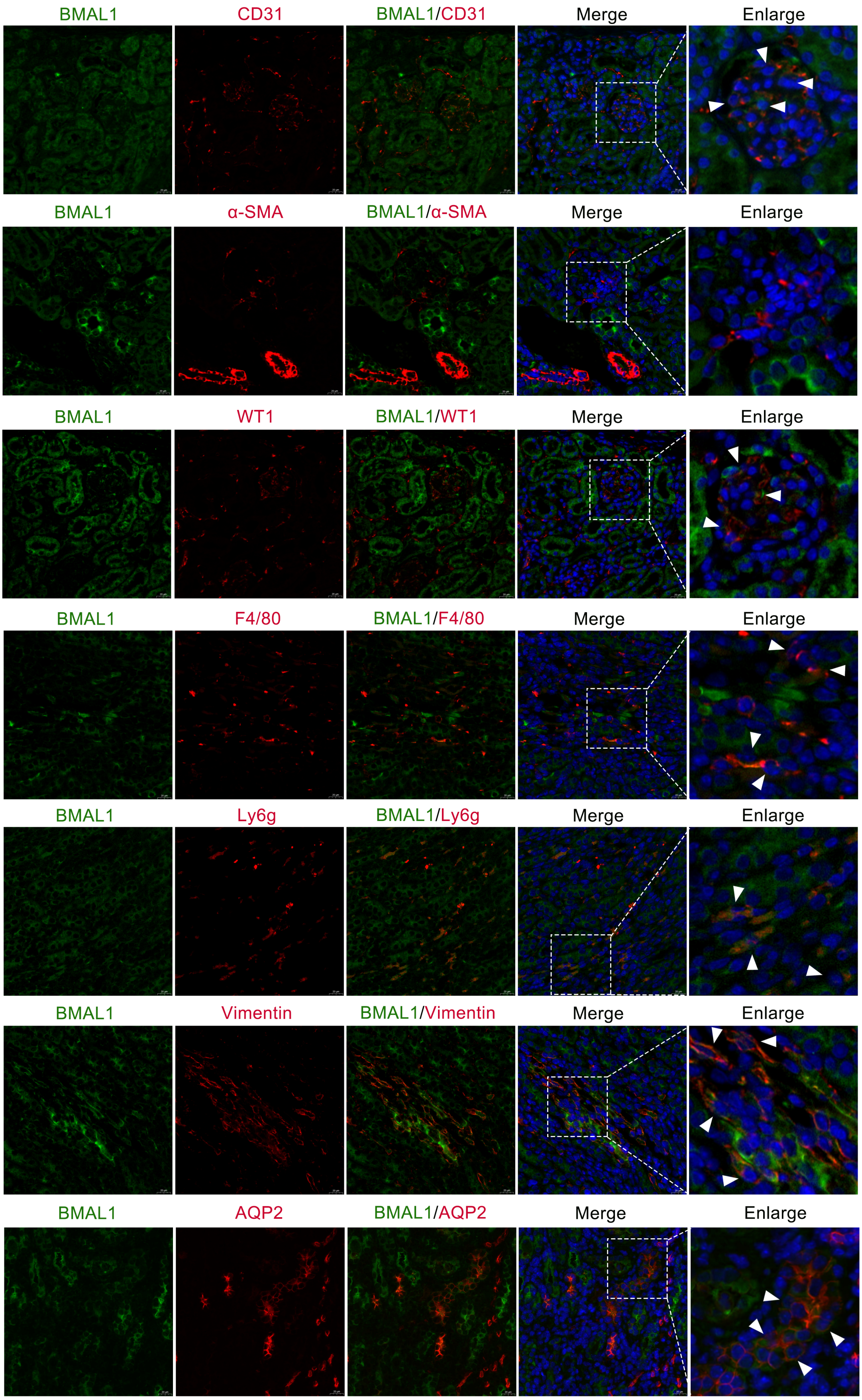

Figure S2. Expression of *BMAL1* in multiple cell types within the kidney. A:

Representative immunofluorescence staining shows the expression of *BMAL1* in glomerular endothelial cells (*CD31*-positive), glomerular mesangial cells (*α-SMA*-positive), glomerular podocytes (*WT1*-positive), macrophages (*F4/80*-positive), neutrophils (*Ly6g*-positive), fibroblasts (*Vimentin*-positive), and collecting duct cells (*AQP2*-positive). White arrows indicate co-localized cells. Scale bars = 20 μm.

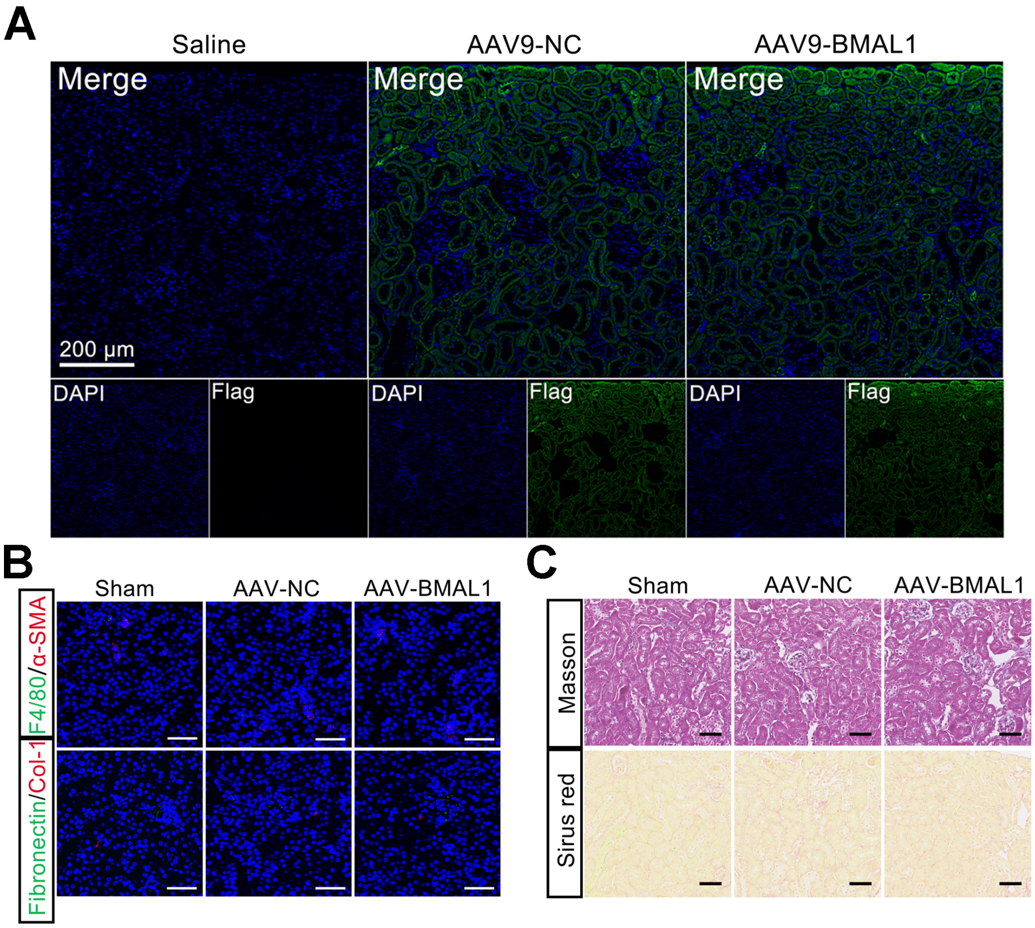

Figure S3. Adenovirus infection alone did not cause MMT or renal fibrosis. A: The success of AAV9 transduction was determined by fluorescence detection. Scale bars = 200 μm. **B-C:** Representative images of immunofluorescence **(B)**, Masson's trichrome and Sirius red staining **(C)** showed the MMT cells and collagen fibre deposition in the kidneys of healthy wild-type C57BL/6 mice after adenovirus infection alone. Scale bars = 50 μm.

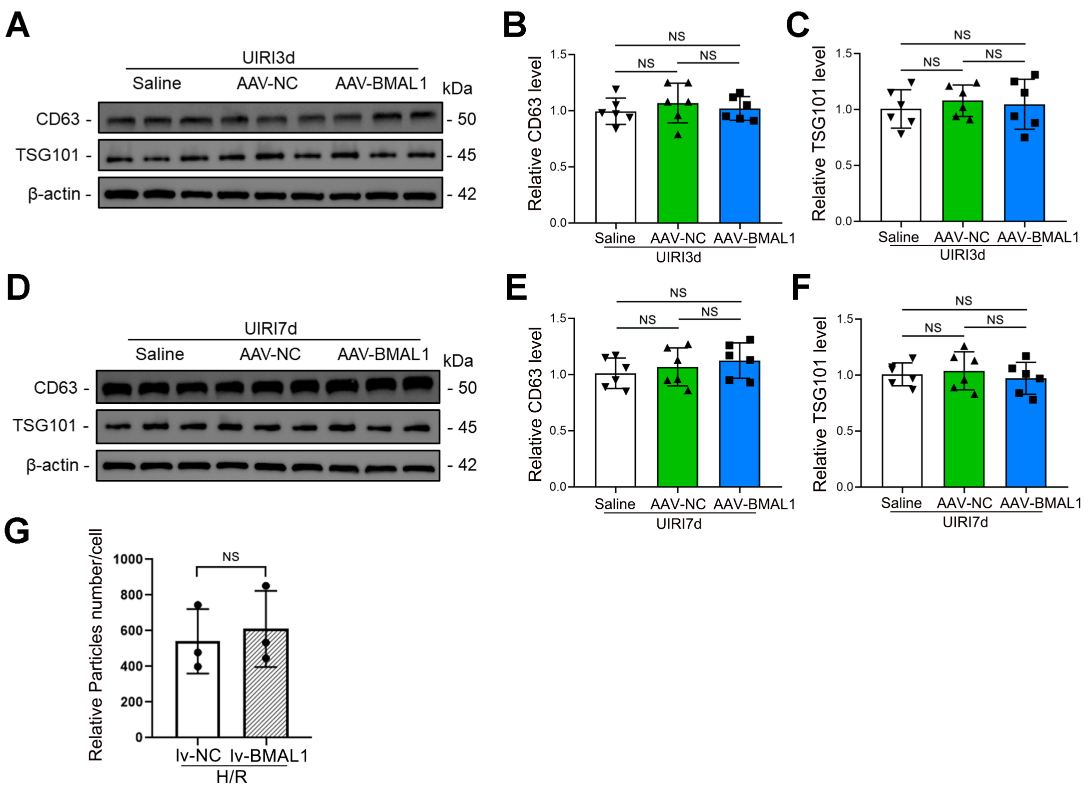

Figure S4. Upregulation of *BMAL1* did not affect the release of exosomes. A-F: Representative western blot banding **(A, D)** and quantitative analysis **(B, C, E, F)** showed the expression levels of *CD63* and *TSG101* in mouse kidneys after AAV-*BMAL1* injection. **G:** The estimated extracellular vesicle secretion quantity of renal tubular epithelial cells. NS, not significant.

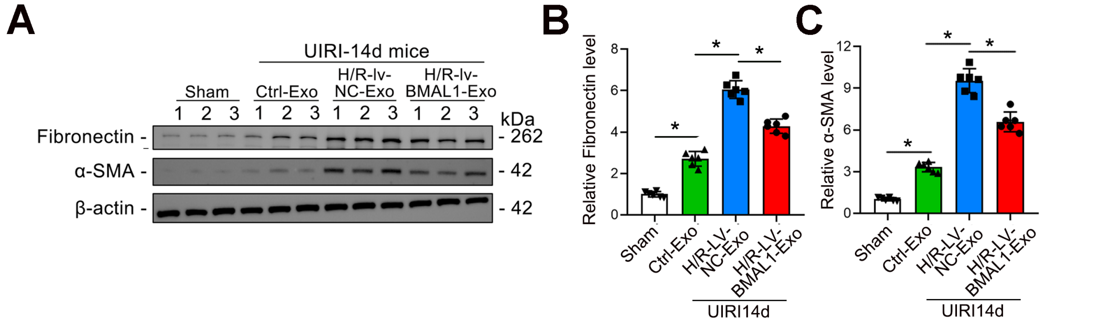

Figure S5. *BMAL1* overexpression mitigated tubular epithelial-derived exosome-mediated MMT and renal fibrosis. A-C: Representative western blot banding **(A)** and quantitative analysis **(B, C)** showed the expression levels of fibronectin and *α-SMA* in mouse kidneys after exosome injection. *P < 0.05.

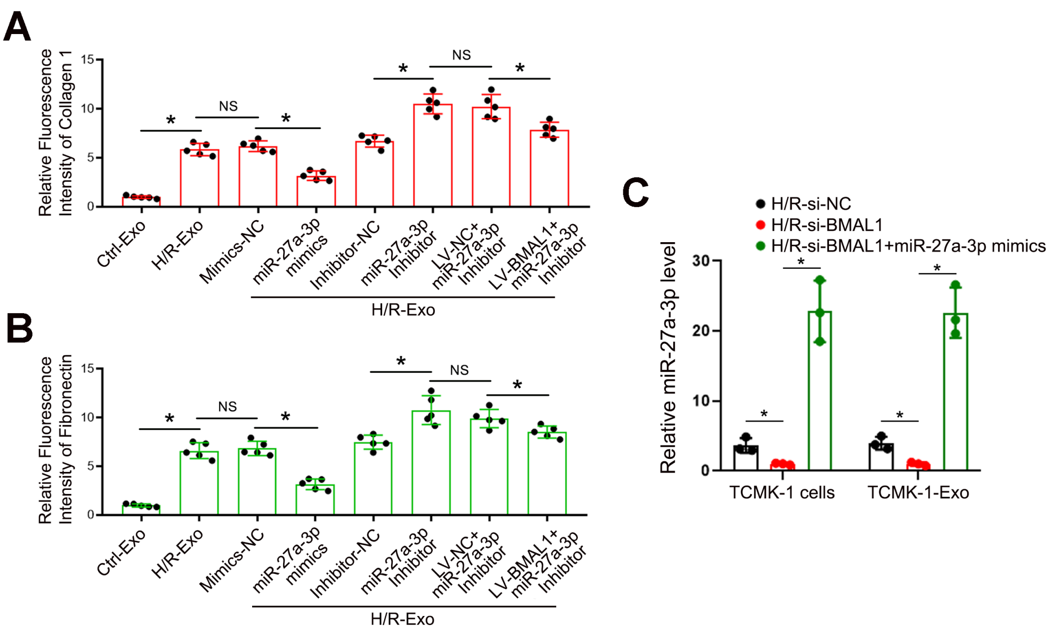

Figure S6. The exosomal *miR-27a-3p* level can directly affect the expression levels of fibronectin and collagen 1 in BMDMs. A, B: Quantitative immunofluorescence staining analysis of collagen 1 **(A)** and fibronectin **(B)** expression in BMDMs stimulated with different groups of TCMK-1-derived exosomes. **C:** qPCR results demonstrated the levels of *miR-27a-3p* in TCMK-1 cells and exosomes in the H/R-si-*NC*, H/R-si-*BMAL1*, and H/R-si-*BMAL1*+ *miR-27a-3p* mimics groups. *P < 0.05. NS, not significant.
